# Biotechnological Advancements in Ovine Bioactives: Mapping Protein Expression Trends and Immuno-Lipidomic Dynamics for Functional Food Applications

**DOI:** 10.3390/ijms27167252

**Published:** 2026-08-14

**Authors:** Kostas A. Triantaphyllopoulos, Kyriakos Bastas, Nikolaos Moustridis, Maria Masoura, Theofilos Massouras

**Affiliations:** 1 Department of Biotechnology, Laboratory of Genetics, School of Applied Biology and Biotechnology, Agricultural University of Athens, 75 Iera Odos Street, 11855 Athens, Greece; 2 Department of Food Science and Human Nutrition, Laboratory of Dairy Research, Agricultural University of Athens, 75 Iera Odos Street, 11855 Athens, Greecetheomas@aua.gr (T.M.)

**Keywords:** breed, Greek sheep, colostrum, lactoferrin, lipogenesis, immunoglobulins, secretome, chemometrics, antagonistic pleiotropy, cardio-protective index

## Abstract

This study explores the biotechnological applications of the ovine secretome by mapping protein expression trends and lipidomic dynamics in Chios (*n* = 5) and Karagouniko (*n* = 10) ewes during early lactation (1, 3, 5, 9, and 15 days post-partum-DPP). Immunoglobulin-G (IgG) expression was monitored via ELISA, lactoferrin (Lf) profiles were quantified using densitometric Tricine-SDS-PAGE, and fatty acid (FA) patterns were resolved by gas chromatography. Two-way mixed-factor repeated-measures ANOVA and Principal Component Analysis (PCA) revealed a highly significant temporal transition (*p* < 0.0001) across all components. Total IgG expression dropped sharply from DPP 1 (62.0–83.5 mg/mL) to day 15 (12.8–21.4 mg/mL), displaying a significant breed-by-time interaction (*p* < 0.0001), while the resilient Karagouniko breed exhibited a more buffered clearance slope. Conversely, local Lf mucosal expression followed a parallel, breed-independent temporal decay trajectory (*p* = 0.5877). Concurrently, saturated FAs decreased while monounsaturated and polyunsaturated fractions rose (*p* < 0.01), optimizing health-related lipid indices over time, including Atherogenicity (IA), Thrombogenicity (IT), and Health-Promoting (HPI). Unsupervised PCA successfully isolated early colostrum profiles based on coordinated immunological and lipidomic vectors. These findings demonstrate that tracking early secretome trajectories serves as a valuable, non-invasive indicator for translational veterinary medicine, establishing these genotype-specific fractions as potential substrates for future evaluation in bioactive functional food matrices research.

## 1. Introduction

The early post-partum mammary secretome represents a highly dynamic, tightly programmed biological fluid that bridges prenatal and life in mammals, orchestrating the vital transition from uterine dependency to autonomous survival [1,2,3]. Unlike mature milk, colostrum is characterized by rapid, time-dependent compositional fluctuations within the first days post-partum, executing a structured metabolic transition into intermediate phases before stabilizing into mature milk within approximately 5–7 days after birth [1,4]. This specialized secretion is critically essential for neonatal survival, particularly in species possessing an epitheliochorial placenta—such as sheep—where the strict syndesmochorial barrier completely prevents the prenatal placental transfer of maternal immunoglobulins to the fetus [5,6,7].

Ovine colostrum is of profound scientific and biotechnological interest due to its exceptionally dense concentration of bioactive macromolecules compared to bovine and caprine counter-matrices [5,8]. Because neonatal lambs are born agammaglobulinemic, they rely entirely on the immediate ingestion and intestinal absorption of maternal antibody fractions, predominantly Immunoglobulin G (IgG), within the first hours of life [6]. The efficiency of macromolecular transport across the neonatal gut epithelium peaks immediately at birth and declines precipitously within 12–24 h due to enterocyte maturation and intestinal closure [9]. Consequently, inadequate colostrum consumption or delayed intake leads to the Failure of Passive Transfer (FPT), a critical clinical condition strongly linked to systemic neonatal morbidity and elevated pre-weaning lamb mortality rates [10,11,12,13].

Beyond systemic immunoprotection via IgG, the ovine secretome is enriched with a sophisticated array of protective glycoproteins and antimicrobial peptides that govern the local neonatal mucosal environment. Among these, Lactoferrin stands out as a highly studied, multifunctional iron-binding glycoprotein that exerts potent antibacterial, antiviral, and immunomodulatory effects, suppressing pathogens in the gut while concurrently regulating local inflammatory responses [14,15,16,17,18]. Lactoferrin serves as a biomarker for COVID-19 [15,19], inflammatory bowel disease (IBD) [20], neurological disorders and aging [21]. In addition, Lf participates in numerous physiological and protective functions, notably antimicrobial and antiviral activities, regulation of cell proliferation and differentiation, modulation of cytokine production, control of inflammatory responses, and immunomodulation [22]. Nevertheless, the cellular and molecular mechanisms underlying Lf’s regulation of inflammatory, antiviral, and immune processes remain incompletely understood.

This protective matrix works in tandem with localized growth factors—including epidermal growth factor (EGF), insulin-like growth factors (IGF-I and IGF-II), and transforming growth factor-beta (TGF-β)—which actively stimulate gastrointestinal epithelial proliferation and cellular maturation [14]. Notably, the early postnatal stage, coinciding with the gut microbiota colonization are critical for the manifestation of the inflammation-associated pathologies of the gastrointestinal tract later in life [23,24]. These bioactive protein concentrations are heavily front-loaded in the initial secretome and experience a sharp, rapid decline within 48–72 h post-partum [9,25].

Simultaneously, the nutritional and lipidomic architecture of sheep colostrum undergoes a massive biochemical reorganization during this narrow transitional window. The early secretome contains exceptionally high gross energy values driven by fat and total protein fractions, paired with low lactose concentrations to accommodate the neonate’s immediate thermoregulatory requirements [26,27,28]. Crucially, the fatty acid profile changes dynamically across the colostrum-to-milk continuum. This transition involves a shift away from the systemic mobilization of preformed, long-chain structural fatty acids derived from maternal adipose tissue towards the activation of localized de novo lipogenesis within the mammary gland, modifying the short- and medium-chain fatty acid distributions [29,30,31,32].

While post-partum temporal dynamics represent the primary driver of these sweeping molecular changes [33,34], the overarching secretome composition is highly modulated by maternal factors, including parity, nutrition, and health status [35,36,37]. Most notably, individual breed genetics exert a profound influence on these timelines. In modern dairy science, intensive human-driven directional selection for high milk volume can inadvertently introduce genetic constraints—such as potential genetic trade-offs—where selection for high milk yield may inversely correlate with structural udder longevity and synchronized immune transport consistency [38]. Consequently, investigating the specific physiological divergence between improved, high-yielding genotypes and resilient, indigenous unselected breeds is essential to understand the modern limits of livestock optimization [39].

In this context, evaluating two distinct ovine breeds (*Ovis aries*) subjected to contrasting human-driven selection pressures—the highly improved, high-yielding Chios breed versus the unselected, hardy Karagouniko breed—provides an ideal intra-species model to isolate the micro-evolutionary impact of directional selection on maternal immune clearance and lipogenic programming. Crucially, this intra-species framework eliminates the confounding inter-species physiological variations that typically complicate cross-species comparisons (e.g., sheep vs. goats).

Concurrently, this rich molecular matrix has attracted significant interest from bystander disciplines, particularly human clinical nutrition and functional food design. The concentration profiles of ovine bioactive fractions, combined with their highly favorable cardio-protective index metrics, position sheep colostrum as a potential biological matrix for functional food research [27]. However, comprehensive field studies utilizing high-resolution quantitative profiling and advanced multivariate chemometrics to map the exact temporal trajectories of these discrete lipid and protein components across distinct indigenous genetic backgrounds remain remarkably scarce.

To address this gap, the present study aims to investigate the high-resolution temporal changes in IgG concentration, mucosal lactoferrin content, and fatty acid structural profiles within Greek sheep cohorts during the critical first two weeks post-partum. By implementing a rigorous chemometric framework, this work seeks to decode the intricate biochemical boundaries of the colostrum-to-milk transition, providing clear, actionable insights for both veterinary optimization strategies and high-value biotechnological food science applications.

## 2. Results

### 2.1. Comparative Expression Kinetics of Whey Immunological and Mucosal Proteins

#### 2.1.1. Temporal Dynamics and Genotypic Divergence of Absolute IgG Secretion

To evaluate the comparative chronological profiles of maternal immune transfer, a two-way mixed-factor repeated-measures ANOVA (RM-ANOVA) was performed to model absolute immunoglobulin G (IgG) clearance patterns across the 15-day postpartum timeline. The statistical framework revealed a highly coordinated physiological sequence characterized by an extremely significant Breed X Time interaction effect (F(4, 52) = 14.87; *p* < 0.0001), which accounted for 4.53% of the continuous biological variance. This critical interaction establishes that the rate, depletion trajectory, and clearance slope of IgG transitioning from dense early colostrum into mature milk depend fundamentally on the maternal genotype. The principal driver of variance within the model was the main effect of Time (F(4, 52) = 292.50; *p* < 0.0001), which explained 89.14% of the total biological shift, while the main effect of Breed was also validated as highly significant (F(1, 13) = 12.89; *p* = 0.0033), accounting for 2.68% of independent variation.

**Figure 1:**
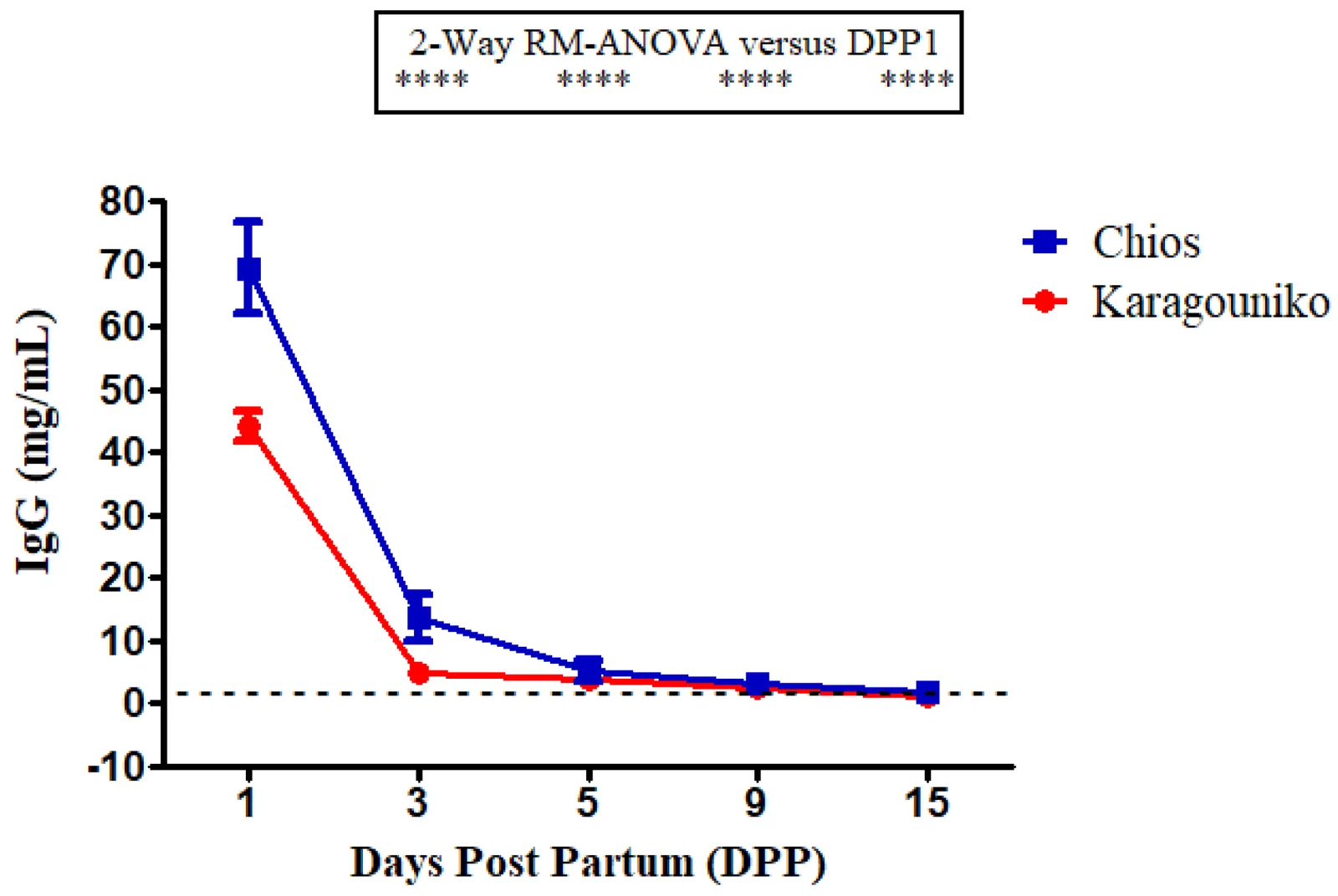
Postpartum depletion kinetics of colostrum whey Immunoglobulin G (IgG) concentration in Chios and Karagouniko ewes. Colostrum profiles across early lactation (DPPs 1, 3, 5, 9, and 15 dpp) reveal a highly significant Breed X Time interaction (F(4, 52) = 14.87, **** *p* < 0.0001, accounting for 4.53% of total variance). The main effects of Time (F(4, 52) = 292.50, **** *p* < 0.0001) and Breed (F(1, 13) = 12.89, *p* = 0.0033) are also highly significant, indicating divergent breed-dependent clearance patterns during the colostrum-to-milk transition. Notes: Chios, Karagouniko sheep breeds.

As illustrated in Figure 1, both ovine populations reached their maximum absolute IgG concentrations on DPP 1 (raw colostrum). However, their clearance rates diverged sharply during the subsequent transitional phase. In the high-yielding Chios breed, initial colostrum IgG concentrations peaked at a mean value of 69.29 ± 7.33 mg/mL before undergoing an immediate, steep depletion to 12.8 mg/mL by DPP 3, thereafter establishing a highly stabilized, low-level steady-state baseline through DPP 15. Conversely, the resilient, indigenous Karagouniko breed displayed a more buffered and sustained depletion curve, starting at a baseline maximum of 44.06 ± 2.37 mg/mL on DPP 1 and maintaining a more gradual downward slope between DPP 3 and DPP 9 before levelling off. This divergence validates a breed-dependent delivery window for passive immunity.

Post-hoc multiple pairwise comparisons confirmed that from DPP 5 onward, intra-breed IgG concentrations stabilized and did not display significant directional variance up to DPP 15 (*p* > 0.05). By DPP 15, absolute IgG levels across both genotypes dropped below the critical 2 mg/mL threshold, statistically marking the formal, absolute completion of the colostrum-to-milk transition within these secretomes.

The mean IgG concentration in the first milking was 44.06 ± 2.37 mg/mL for Karagouniko breed and 69.29 ± 7.33 mg/mL for Chios breed, showing marked inter-individual variability. A sharp decline was observed during the early postpartum period, with the most pronounced reduction occurring between DPP 1 and DPP 3.

From day 5 onwards, IgG concentrations did not differ significantly up to day 15 postpartum in both breeds. By DPP 15, IgG levels decreased to below 2 mg/mL, indicating a complete transition from colostrum to mature milk secretion.

#### 2.1.2. Multivariate Modeling of Breed-Specific IgG Heterogeneity

**Figure 2:**
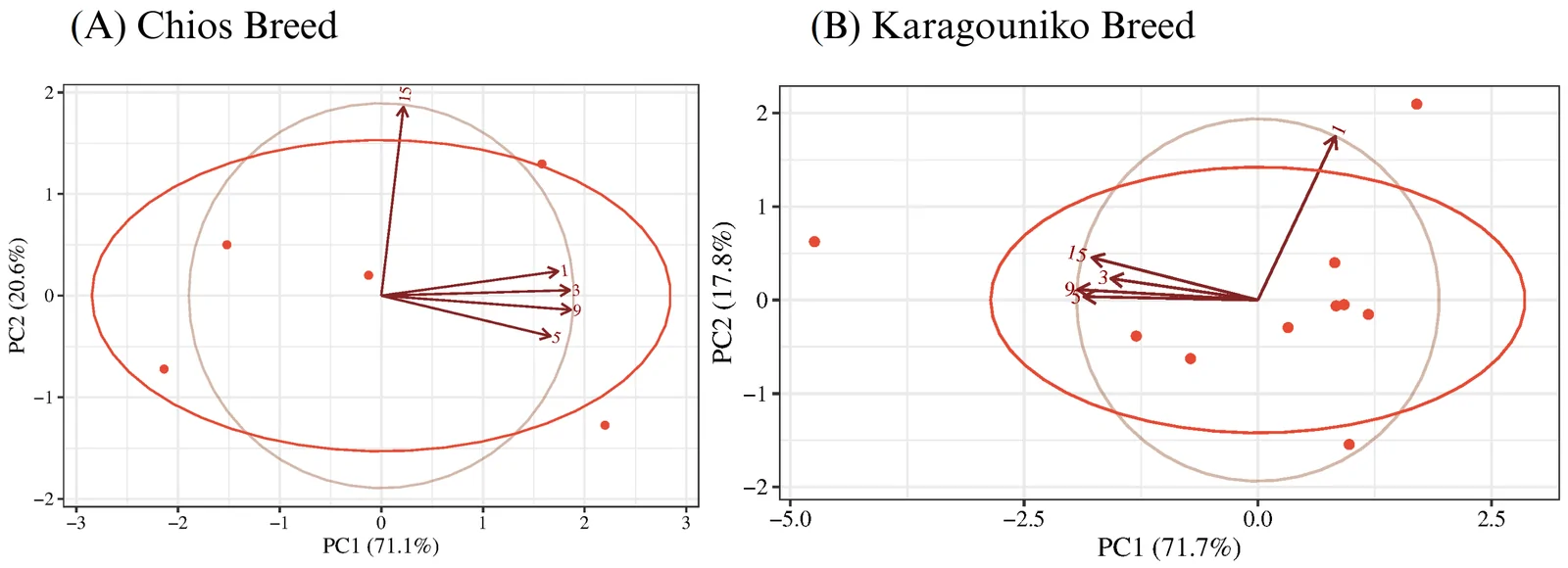
Multivariate relationship between breed and temporal IgG concentration in colostrum secretion (details in the text).

To evaluate the standalone longitudinal trajectories and internal variance structure of maternal total immunoglobulin G (IgG) expression, separate unsupervised Principal Component Analysis (PCA) biplot models were generated for each breed (Figure 2). For both genotypes, a two-dimensional projection proved mathematically exceptional, accounting for 91.7% (PC1 = 71.1%, PC2 = 20.6%) of the cumulative variance in the Chios cohort (Figure 2A) and 89.5% (PC1 = 71.7%, PC2 = 17.8%) in the native Karagouniko cohort (Figure 2B). These metrics confirm a remarkably high signal-to-noise ratio within the localized datasets.

The underlying eigenvalue extraction and variance partitioning metrics confirming the mathematical strength of these individual dimensions are documented in Table S1. The mathematical validity of restricting both models to two dimensions was further supported by the explained variance (or scree) plot analysis (Figure S1), which highlighted sharp, matching inflection points after the second principal component (Figure S1, Chios and Karagouniko Breed IgG Variance in the Supplementary Materials).

To evaluate the architectural stability and population distribution boundaries within these models, two layers of geometric ellipses were applied. The centered, neutral-coloured circular rings indicate the overall model limits, while the oriented, coloured rings represent the calculated 95% data confidence ellipses for each sample set. These 95% confidence ellipses mathematically define the true multidimensional variance zone of each breed; tighter, more compressed ellipses reflect highly synchronized individual trajectories, whereas elongated axes reveal the primary direction of biological change across time. The complete principal component loading coefficients and rotation matrix weights across all five extracted principal components for individual sampling intervals are catalogued in Table S2.

In the Chios breed (Figure 2A), the loading vectors for days post-partum (DPP) 1, 3, 9, and 5 cluster along the positive horizontal axis of PC1, indicating a highly uniform, parallel progression during early colostrum production and early transitional phases.

This core timeline is counterbalanced by the vertical loading vector of mature milk at DPP 15, which drives strongly upward into the positive PC2 quadrant, pulling the sample scores along a distinct maturation path. Conversely, the indigenous Karagouniko breed (Figure 2B) demonstrates an altered directional configuration. The markers for transitioning and mature milk (DPP 3, 9, and 15) stretch along the negative horizontal PC1 axis, while the immediate colostrum secretome at DPP 1 tracks upward into the positive PC2 quadrant. 

Separate Principal Component Analysis (PCA) biplot configurations detailing the longitudinal trajectories of maternal colostrum and milk Immunoglobulin G (IgG) expression for (A) the Chios genotype (*n* = 5) and (B) the native indigenous Karagouniko genotype (*n* = 10) across five post-partum intervals (1, 3, 5, 9, and 15 DPP). Directional dark red arrows represent the loading vectors for individual sampling days. The neutral-coloured concentric circles centered at the origin denote overall model limits, whereas the coloured, oriented rings define the 95% data confidence ellipses for each animal cohort. The models capture 91.7% and 89.5% of cumulative experimental variance for Chios and Karagouniko, respectively, demonstrating clear differences in chronological transition velocity between the two genotypes.

The distinct orientations and structural shapes of these confidence ellipses highlight that although both breeds undergo an extensive systemic transition from colostrum to mature milk, the internal pacing, regulatory switches, and temporal shifts of their protective IgG secretomes remain fundamentally distinct and breed-specific.

### 2.2. Electrophoretic Micro-Quantification and Depletion of Ovine Colostrum Lactoferrin

**Figure 3:**
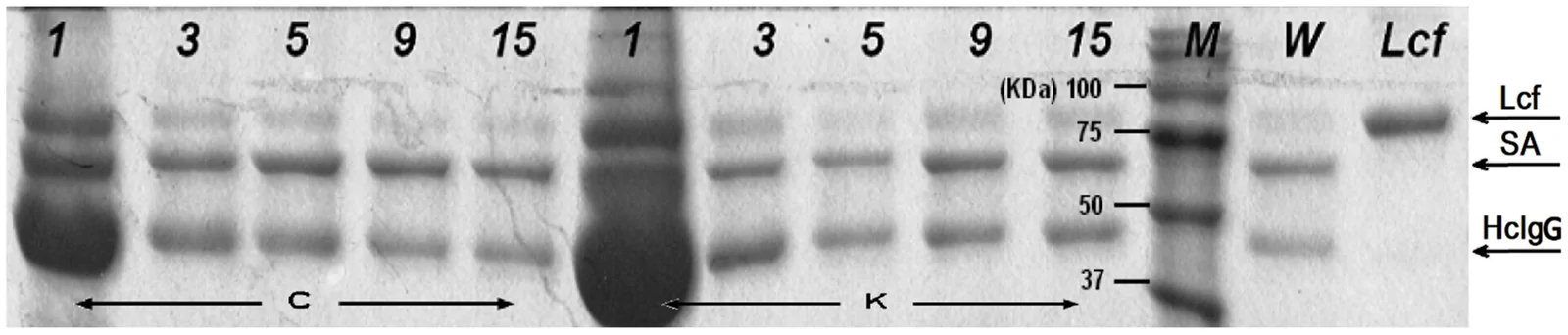
Representative sodium dodecyl sulfate–polyacrylamide gel electrophoresis (SDS-PAGE) profiles of sheep colostrum whey proteins during the transition to mature milk. Resolution was performed using a Tricine SDS-PAGE system under reducing conditions. Equal protein concentrations were loaded into each lane, with the exception of lanes C1 and K1, where protein loading was doubled to ensure optimal signal intensity for subsequent standard curve interpolation. Lanes correspond to: C1, C3, C5, C9, C15: Colostrum whey fractions from Chios ewes at days post-partum (DPP) 1, 3, 5, 9, and 15, respectively. K1, K3, K5, K9, K15: Colostrum whey fractions from Karagouniko ewes at 1, 3, 5, 9, and 15 DPP, respectively. Notes: Lcf, lactoferrin; SA, serum albumin; HcIgG, heavy chain immunoglobulin G; M, high-resolution molecular weight marker; W, colostrum whey fraction; C, Chios breed; K, Karagouniko breed; DPP, days post-partum.

Densitometric evaluation via Tricine–Sodium Dodecyl Sulfate–Polyacrylamide Gel Electrophoresis (Tricine–SDS–PAGE) of individual whey fractions resolved a highly abundant, distinct molecular band corresponding to mucosal Lactoferrin (Lf) during the early post-partum hours, followed by a progressive chronological depletion (Figure 3). As shown in Figure 3, the individual colostrum whey fractions successfully resolved clear, highly abundant polypeptide bands corresponding to maternal serum albumin (SA; 66 kDa), the heavy chain of immunoglobulins (HcIgG; 50 kDa), and mucosal-type Lactoferrin (Lf) migrating at approximately 78 kDa (Figure 3).

Furthermore, visual inspection of the gel lanes highlights a striking chronological reduction in band density across the 15-day postpartum timeline for both populations, marking the steady biochemical transition of raw immune secretome into standardized mature milk fractions.

Overall, lactoferrin showed a biphasic pattern characterized by a rapid early decline followed by a low-concentration plateau phase, as lactoferrin levels stabilized at low but detectable concentrations up to DPP 15, consistent with the physiological transition from immune-protective secretion to mature milk. Thus, this decline was reminiscent to colostrum IgG with a similar but slightly less abrupt temporal pattern.

#### Synchronized, Breed-Independent Clearance Kinetics

**Figure 4:**
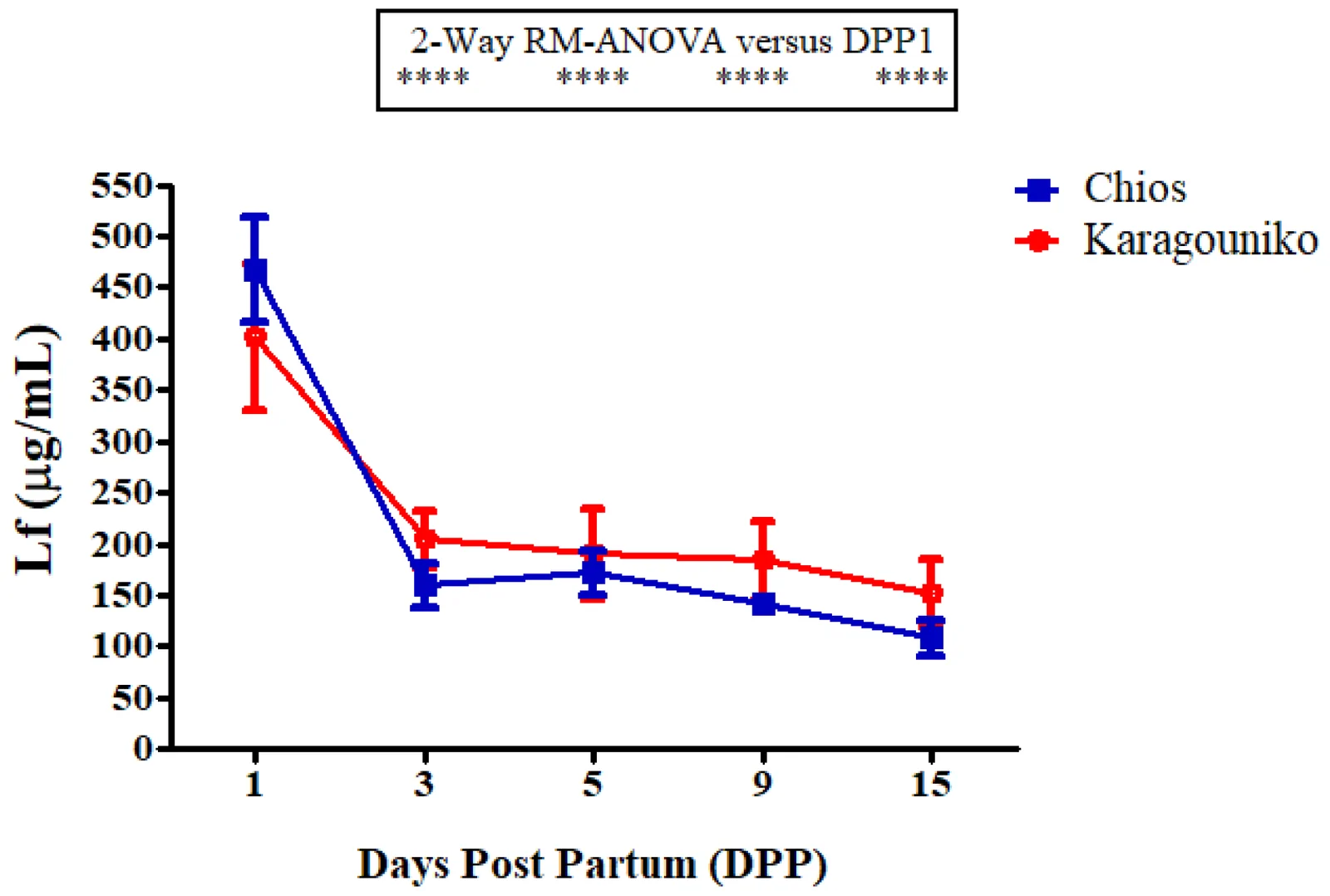
Postpartum depletion kinetics of colostrum whey Lactoferrin (Lf) in Chios and Karagouniko ewes. Two-way RM-ANOVA confirms a completely non-significant Breed X Time interaction (F(4, 24) = 0.72, *p* = 0.5877, accounting for only 2.63% of total variance) and a non-significant main effect for Breed (*p* > 0.05). Conversely, the downward trajectory is governed exclusively by the effect of Time, which explains 71.56% of the total biological variance (F(4, 24) = 19.57, **** *p* < 0.0001, 95% confidence intervals). Values represent Mean ± SEM. Post-hoc multiple pairwise comparisons are benchmarked against the DPP 1 baseline reference column; asterisks denote significant differences (**** *p* < 0.0001).

Following digital densitometry validation of these targeted 78 kDa bands, absolute concentration shifts (μg/mL) were modeled mathematically, i.e., plotted on a graph versus days post-partum (1, 2, 5, 9 and 15 DPP) (Figure 4).

In direct contrast to the divergent clearance profiles observed for systemic maternal IgG, absolute Lactoferrin levels adhered to a highly uniform, parallel depletion pathway across both genetic lines. For both breeds, colostrum Lactoferrin levels on DPP1 drop immediately and identically by DPP3, continuing along a synchronized baseline through DPP 15.

Two-way mixed-factor RM-ANOVA confirmed that the Breed X Time interaction effect was completely non-significant, explaining a negligible 2.63% of total variance (F(4, 24) = 0.72, *p* = 0.5877). Furthermore, the main effect of Breed exerted no structural influence on the model (F(1, 6) = 0.71, *p* = 0.4317, accounting for just 0.41% of absolute variance), proving that baseline mucosal lactoferrin expression tracks identically between Chios and Karagouniko ewes.

Instead, Lactoferrin clearance is governed exclusively by the main effect of Time, which accounted for 71.56% of the total biological variance within the secretome (F(4, 24) = 19.57, *p* < 0.0001, 95% confidence intervals). As demonstrated in Figure 4, the initial colostrum peak at DPP 1 (467.5 μg/mL for Chios and 402.1 μg/mL for Karagouniko) underwent an immediate, synchronized drop by DPP 3 (159.2 μg/mL and 206.4 μg/mL, respectively). Bonferroni post-hoc multiple pairwise comparisons benchmarked against the DPP 1 baseline confirmed that this sharp early depletion was highly significant across all subsequent column pairs (**** *p* < 0.0001), with concentrations transitioning into a flat, low-level steady-state mucosal plateau from DPP 5 through the final mature milk validation at DPP 15.

### 2.3. Fatty Acid Composition and Profile Dynamics

Significant changes were observed in the total fatty acid composition during the transition from colostrum to mature milk. Early colostrum was characterized by higher proportions of medium-chain fatty acids (MCFAs), particularly C10:0 and C12:0, whereas long-chain fatty acids (LCFAs), mainly C16:0 and C18:1, increased progressively over time. Saturated fatty acids (SFAs) dominated the early postpartum period, while unsaturated fatty acids increased gradually. By day 15 postpartum, both breeds exhibited a lipid profile characteristic of mature milk, although with distinct temporal dynamics.

Breed-specific differences were evident. In Chios breed, short-chain fatty acids (SCFAs; C4:0, C6:0, C8:0) increased over time, whereas in Karagouniko sheep, SCFAs remained relatively stable. Medium-chain fatty acids (C10:0, C12:0, C14:0) decreased significantly in Chios, while Karagouniko showed a more variable pattern, including an increase in C10:0 between DPP 5 and 15. Oleic acid (C18:1) was consistently the dominant fatty acid in Karagouniko throughout lactation, whereas in Chios it became dominant only after day 3 postpartum. The detailed concentrations expressed as a weight percentage (% *w*/*w*) of total FAMEs across all five timepoints are compiled in Table 1.

#### 2.3.1. Chemometric Profiling of Longitudinal Fatty Acid Profile Trajectories via PCA Biplots

**Figure 5:**
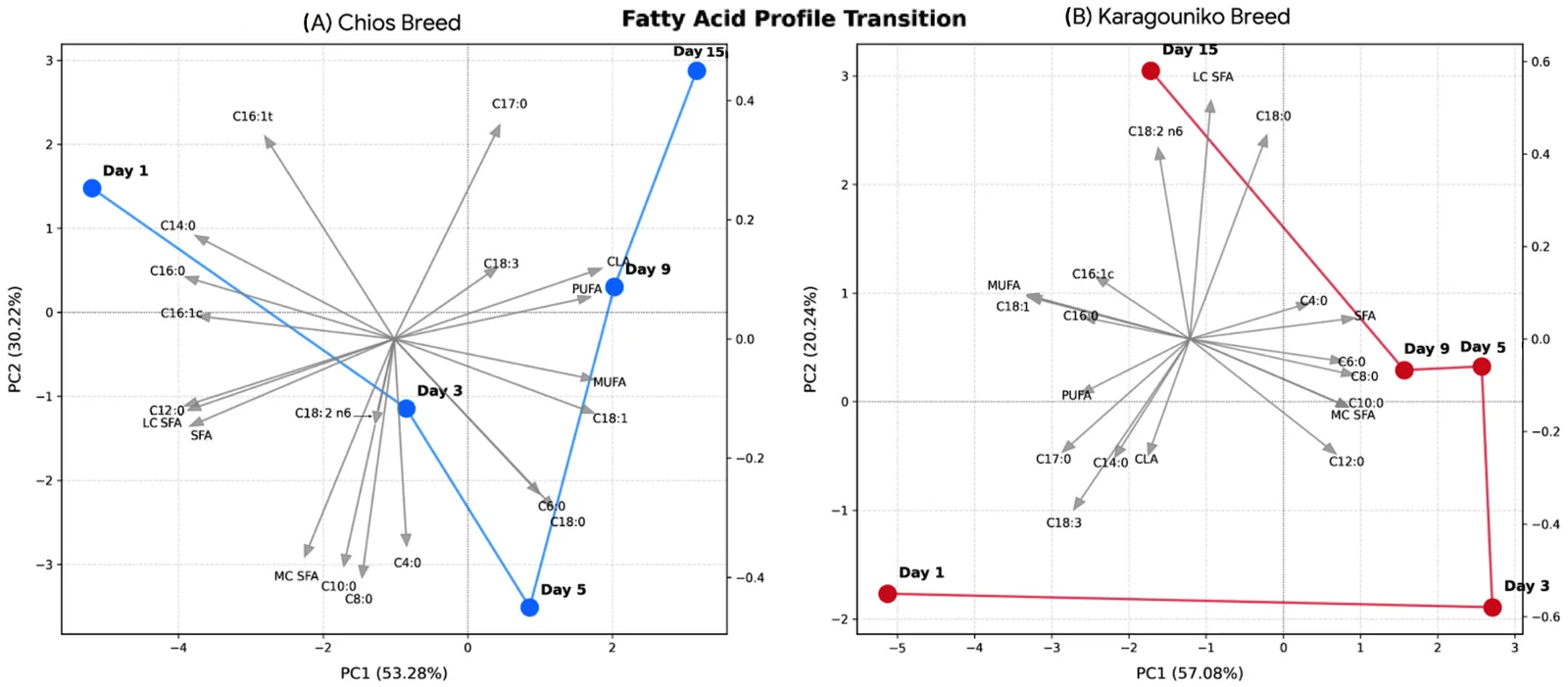
Principal Component Analysis (PCA) biplots characterizing the longitudinal transitions of colostrum sheep fatty acid profiles. Independent biplots are presented for (**A**) the Chios breed (parabolic path) and (**B**) the Karagouniko breed (sequential path) across five distinct lactation time points (DPP 1, DPP 3, DPP 5, DPP 9, and DPP 15). For both panels, primary axes represent sample score positions relative to principal component 1 (PC1) and principal component 2 (PC2), with the percentage of explained variance annotated parenthetically in the X-Y-axes. Overlaid dark grey vector arrows represent the loading coefficients (rotation vectors) of individual fatty acids and aggregate lipid categories (SFA, saturated fatty acids; MC SFA, medium-chain SFAs; LC SFA, long-chain SFAs; MUFA, monounsaturated fatty acids; PUFA, polyunsaturated fatty acids; CLA, conjugated linoleic acid).

To explore the high-dimensional relationships and cross-temporal trajectories of these shifting lipid fractions, unsupervised chemometric modeling was applied. Unsupervised Principal Component Analysis (PCA) successfully mapped the temporal transition of the post-partum secretome, aligning coordinated lipid vectors to demonstrate clear breed-specific divergence (Figure 5) [40,41]. The underlying eigenvalues and variance partition matrices for these longitudinal post-partum colostrum fatty acid profiles are structured in Table S3.

For both the Chios and Karagouniko genotypes, a classic, sharp inflection point or “elbow” is observed immediately following PC2, after which subsequent dimensions contribute minimally to explaining the total variance matrix. Retaining exclusively the first two principal components thus yields a highly optimized, low-dimensional reduction of the longitudinal data that preserves the overwhelming majority of biological signal while effectively filtering out residual stochastic noise. Morevover, the mathematical validity of restricting both models to two dimensions was further supported by the explained variance (or scree) plot analysis (Figure S2), which highlighted sharp, matching inflection points after the second principal component.

For the Chios breed (Figure 5A), the first two principal components captured 83.50% of the total cumulative variance, with PC1 accounting for 53.28% and PC2 accounting for 30.22%. The score plot maps a distinct, highly pronounced parabolic trajectory across time. Days post-partum (DPP) 1 and DPP 3 colostrum and transitional samples clustered distinctly on the negative side of the PC1 domain, a placement strongly driven by long-chain saturated fatty acids (SFA, including C12:0, C14:0, and C16:0). As lactation progressed toward mature milk at DPP 9 and DPP 15, the sample scores transitioned smoothly into the positive quadrants of both PC1 and PC2. This advanced mature milk phase was heavily characterized by loading vectors for monounsaturated fatty acids (MUFA), polyunsaturated fatty acids (PUFA), oleic acid (C18:1), and conjugated linoleic acid (CLA). Conversely, the short- and medium-chain fatty acids (C4:0, C6:0, C8:0, and C10:0) pointed towards the negative PC2 space, marking the specialized intermediate transitional phase of DPP 3 and DPP 5. The spatial distribution and separation of these axes are explicitly governed by the behavior of the primary fatty acid variables, whose separate principal component loading coefficients and rotation matrix weights are detailed in Table S4a for the Chios breed.

Consistent with these multivariate spatial distributions, continuous longitudinal profiling confirmed that the overall composition of Chios colostrum steadily approaches that of normal, mature milk within fifteen days after parturition and this is also consistent with other studies in goat [42]. However, the underlying fatty acid trends revealed stark differences in accumulation and depletion rates between the two genotypes during the first two weeks post-partum. A statistically significant post-partum upregulation in individual short-chain volatile fatty acids (SCFA; C4:0, C6:0, and C8:0) was observed, reaching a clear maximum concentration during the early transitional timeline (DPP 3 -DPP 5) in the Chios breed, followed by a steady depletion as the mature milk phase stabilized at DPP 15. This early metabolic burst was accompanied by a significant longitudinal decrease in medium-chain saturated fatty acids (MCFA; C10:0, C12:0, and C14:0), leading to a net reduction in total SFA output by the end of the two weeks. In terms of long-chain structural fractions, oleic acid (C18:1) became a prominent, driving component in Chios milk only after the third day post-partum.

For the Karagouniko breed (Figure 5B), the first two principal components explained 77.32% of the total experimental variance, with PC1 explaining 57.08% and PC2 explaining 20.24%.

The specific rotation matrix weights determining this spatial layout for individual fatty acid variables in the Karagouniko model are provided in Table S4b.

In sharp contrast to the parabolic progression observed in the Chios breed, the indigenous Karagouniko ewes displayed a sequential chronological path across time. Early colostrum (DPP 1) was situated in the lower-left quadrant of the coordinate system (PC1 = −5.13, PC2 = −1.77). The lipid profile then shifted across the graph to settle in the far-right quadrants during the transitional stages of DPP 3 (PC1 = +2.71) and DPP 5 (PC1 = +2.57), before completing its trajectory in the upper-left quadrant by the mature milk stage at DPP 15 (PC1 = −1.72, PC2 = +3.05).

The loading vectors show that the intensive intermediate metabolic shift (DPP 3, 5, and 9) was heavily driven by total SFA, medium-chain SFAs (MC SFA), and short-chain volatile fatty acids (C4:0, C6:0, C8:0, C10:0, and C12:0), which pointed sharply to the right. Conversely, the negative PC1 region was defined by MUFA, PUFA, C18:1, and CLA vectors, which directly aligned with the unique, overlapping horizontal positioning of both DPP 1 (colostrum) and DPP 15 (mature milk) on the left side of the biplot. This complex spatial layout perfectly models the empirical observation that the steady post-partum decrease in MCFA and total SFA seen in Chios was not replicated in the Karagouniko breed. Instead, the Karagouniko ewes exhibited a late-stage surge in capric acid (C10:0) from the 5th to the 15th day post-partum, keeping the medium-chain saturated matrix elevated. Long-chain structural fats (C18:0 and LC SFA) were highly elevated along the positive vertical PC2 axis, tracking the final maturation phase at DPP 15. Crucially, oleic acid (C18:1) was maintained as the absolute predominant fatty acid for the Karagouniko breed throughout the entire duration of the study (DPP 1 to 15), closely followed by palmitic acid (C16:0), which represents a major physiological departure from the delayed accumulation trend observed in the Chios breed.

Taken together, these observations highlight a clearly divided pattern in early lactation profiles, characterized either by a sustained elevation of SFAs and LC SFAs, or a progressive transition toward PUFAs, MUFAs, and C18:1. Ultimately, these distinct developmental trajectories suggest that colostrum lipid maturation is dynamically governed by a combination of breed, parity, species-specific programming (extending from domestic ruminants to humans), and environmental factors such as maternal diet [29,43,44,45,46,47,48].

**Figure 6:**
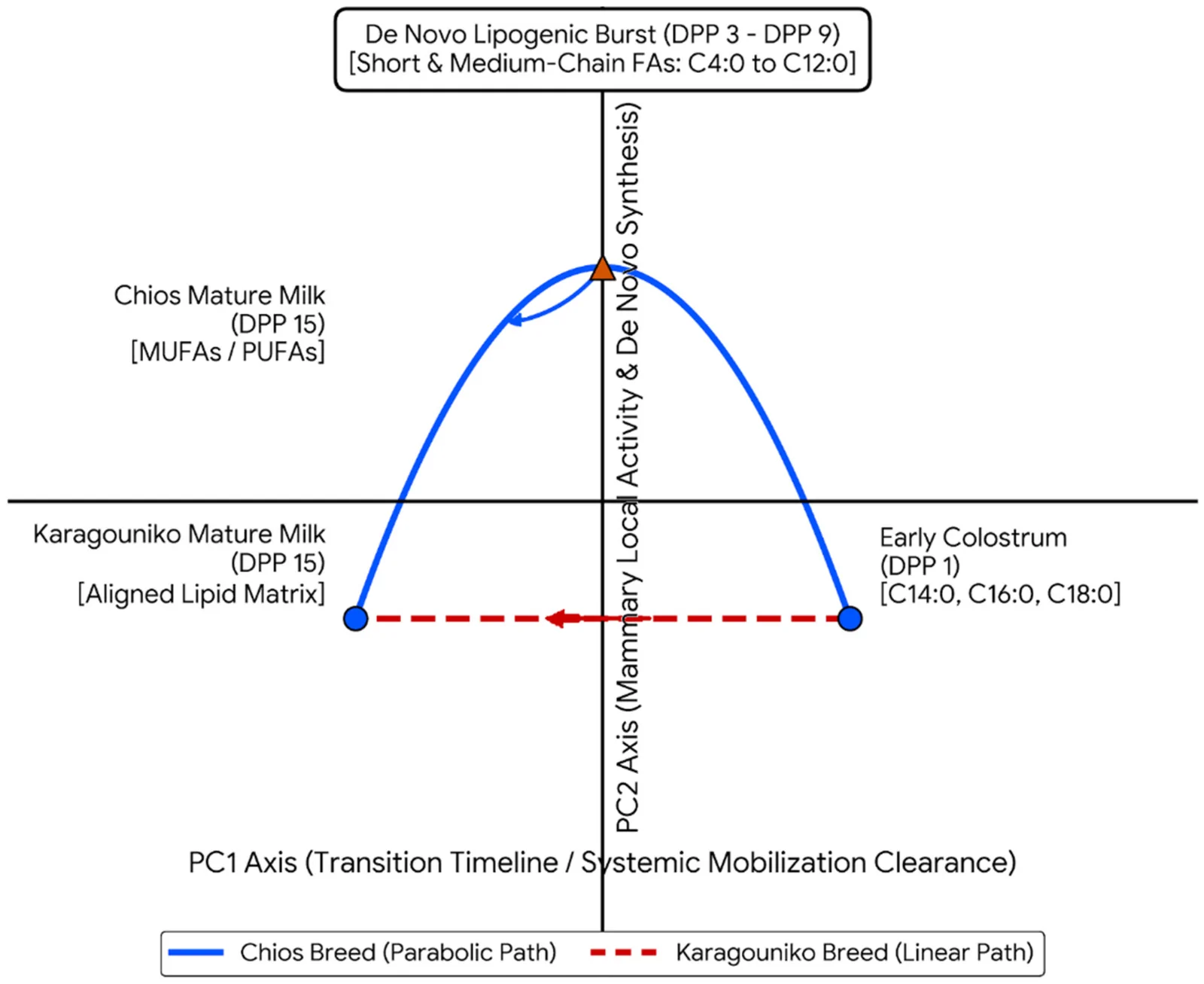
Proposed hypothetical metabolic model of genotype-specific lipogenic programming during the early post-partum transition (DPP 1 to DPP 15) in Chios and Karagouniko ewes. The diagram contrasts the steady, sequential trajectory of systemic adipose tissue mobilization in the indigenous Karagouniko breed with the specialized, parabolic de novo short- and medium-chain fatty acid synthesis burst (C4:0 to C10:0) localized within the mammary epithelial cells of the high-yielding Chios breed. The sloped blue arrow along the Chios parabola serves as a directional trajectory vector, explicitly mapping the chronological kinetics of the secretome across the lactation timeline. The right-to-left orientation of this parabolic path tracks the biological shift from early transitional stages (DPP 3 -5), which cluster on the right due to high concentrations of locally synthesized short- and medium-chain fatty acids, towards mature milk stabilization (DPP 15) on the left, driven by long-chain structural and unsaturated lipid fractions. The parabolic blue curve depicts the initial surge and subsequent shift in de novo fatty acid synthesis characteristic of the Chios breed. The red (right-to-left) positioned beneath the parabolic curve illustrates the sustained, linear reliance of the Karagouniko breed on systemic fat mobilization (non-esterified fatty acids utilization) and steady medium-chain saturated matrix secretion across the sampling period. Note: This conceptual model is formulated from phenotypic fatty acid gas chromatography (GC-FID) profiles and chemometric PCA trajectories; direct gene expression (RT-qPCR) and lipogenic enzyme activity assays were not evaluated.

To visually contextualize the metabolic mechanisms driving the distinct spatial configurations observed in the empirical PCA biplots, a comparative lipogenic pathway proposed hypothetical model was constructed (Figure 6). The chemometric trajectories reveal two divergent modes of metabolic programming during the colostrum-to-milk transition [49].

For the indigenous Karagouniko breed, the trajectory reflects a steady, sustained reliance on systemic lipid sources. Because C18:1 remains the predominant fatty acid uniformly from DPP 1 through DPP 15, followed closely by C16:0, this profile indicates a continuous, stable mobilization of peripheral adipose tissue reserves to sustain early lactation demands without major shifts in baseline mammary synthesis [50]. This directly explains why the Karagouniko breed does not follow the temporal drop in overall SFAs or MCFAs seen in the Chios genotype.

In contrast, the Chios breed exhibits a dynamic metabolic shift along its parabolic biplot trajectory, representing a specialized, biphasic adaptation [40]. During the early post-partum transition (DPP 3 and DPP 5), the Chios profile displays an intense, transient upregulation of short- and medium-chain fatty acids (C4:0 to C10:0). Because these volatile fatty acids cannot be transported from peripheral body fat stores and must be synthesized entirely locally, this early alignment directly indicates an active acceleration of de novo lipogenesis localized within the mammary epithelial cells, driven by key local enzymes such as acetyl-CoA carboxylase (ACC) and fatty acid synthase (FAS) [51]. As the lactation timeline approaches mature milk stability at DPP 15, the trajectory loops away from these vectors, demonstrating that this early metabolic burst subsides. This internal metabolic transition coincides with a synchronized longitudinal drop in MCFAs and overall SFAs, alongside a delayed prominence of C18:1 only after the third day.

This structural model confirms that tracking these distinct spatial movements provides a valuable, non-invasive method to investigate breed-specific metabolic trajectories and secretome dynamic shifts during early lactation.

The composition of sheep colostrum approaches those of normal milk within fifteen days after parturition. The fatty acid analysis in the two sheep breed colostrum show, different trend in their rising or fall during the first 2 weeks (days post partum-DPP):

A statistically significant increase in the short chain fatty acids (SCFA) (C4:0, C6:0, C8:0) in the course of the DPP 15 was observed in Chios breed, while this was not observed in Karagouniko breed.

A significant decrease was observed in Chios breed for the medium chain fatty acids (MCFA) (C10:0, C12:0, C14:0), but this trend was not followed in Karagouniko breed due to the increase of C10:0 from 5th to 15th day (DPP).

SFA shows a decrease in the course of 2 weeks (DPP) in Chios breed, while not the case for Katagouniko breed. C18:1 was the predominant fatty acid for Katagouniko breed throughout the course of the study (1 -15th DPP), followed by C16:0, unlike Chios breed, where this is prominent only after the third day.

**Figure 7:**
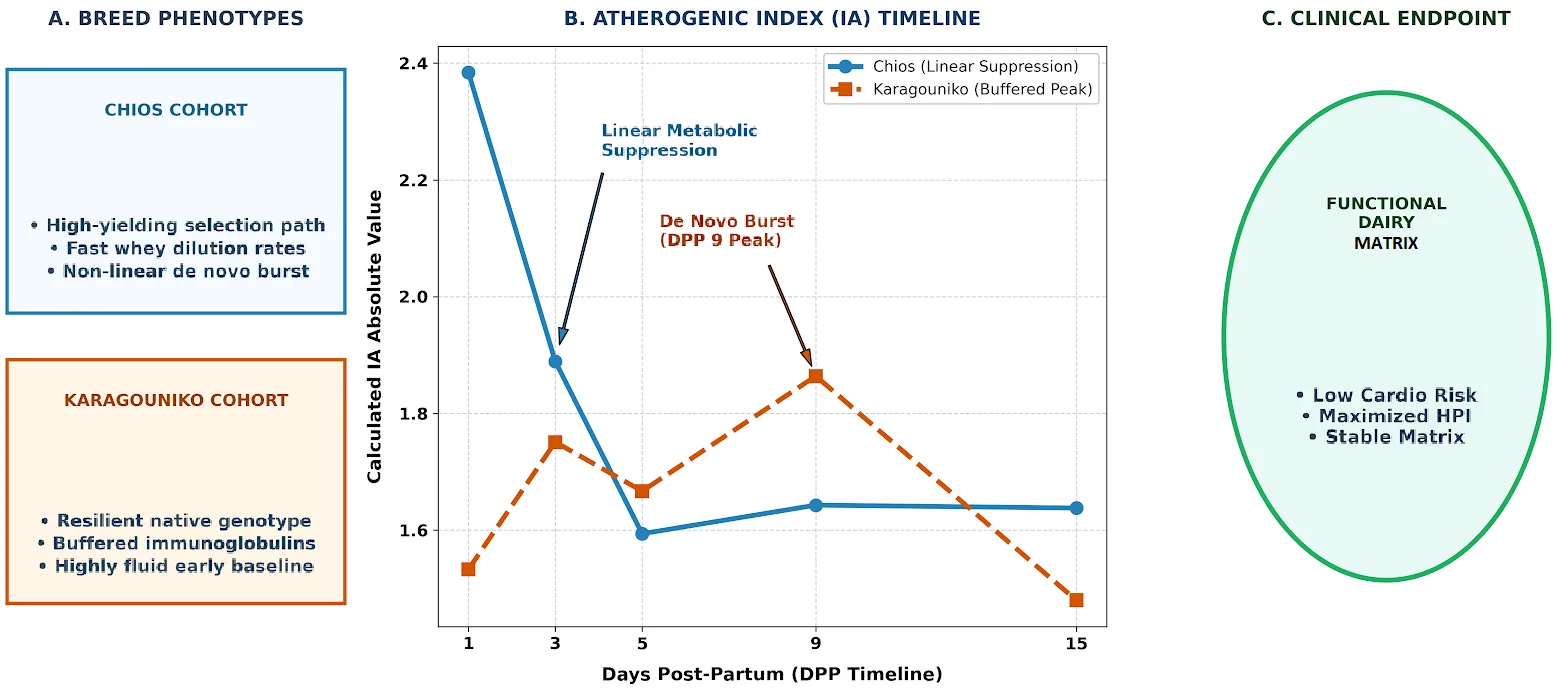
Multi-panel conceptual framework tracking genotype-specific lipogenic programming and lipid quality endpoints in Chios and Karagouniko ewes across the early lactation timeline. Panel (**A**) outlines the distinct physiological and metabolic profiles characterizing the two breed phenotypes; Panel (**B**) tracks the longitudinal evolution of the calculated Atherogenic Index (IA) from day post-partum (DPP) 1 to 15, indicating localized mammary adaptations and systemic regulatory shifts; Panel (**C**) represents potential downstream impacts on functional dairy classifications and human health outcomes.

This complex restructuring of individual lipid fractions directly shapes the overall nutritional safety profile of the secretion. When these changes are integrated into a holistic health indicator, the resulting lipid safety scores reveal an inverse performance dynamic between the cohorts (Figure 7). Specifically, the Chios breed demonstrates a sharp, linear suppression of the Atherogenic Index (IA) during the initial calving window, shifting from an early peak down to a stable baseline. Conversely, the Karagouniko breed presents a highly fluid baseline characterized by a transient index elevation peaking around DPP 9, indicating a delayed or buffered lipogenic stabilization window (Figure 7).

Ultimately, these highly divergent chemometric trajectories demonstrate that while the indigenous Karagouniko breed relies consistently on systemic adipose tissue mobilization to sustain itself through early lactation, the high-yielding Chios breed undergoes an intensive, localized mammary metabolic adaptation characterized by a distinct, chemometrically detected de novo fatty acid synthesis burst.

#### 2.3.2. Integrated Dynamic Modeling of Cardio-Protective, Nutritional Lipidomic and Quality Indices

The structural remodeling of absolute fatty acid classes directly dictates the downstream nutritional quality, clinical health-promoting lipid indices, and atherogenic potential of the secretome across the postpartum timeline.

To comprehensively evaluate the dietary fatty acid impacts of Chios and Karagouniko transitions on human cardiovascular health, the Index of Atherogenicity (AI) and the Index of Thrombogenicity (TI)—originally established by Ulbricht and Southgate (1991) [52]—were calculated alongside the Health-Promoting Index (HPI):

(1) AI = [C12:0 + (4 × C14:0)+ C16:0]/ΣUFA

(2) TI = (C14:0 + C16:0 + C18:0)/[(0.5 × ΣMUFA) + (0.5 × C18:2 n–6) + (3 × C18:3 n–3) + CLA]

(3) HPI = ΣUFA/[C12:0 + (4 × C14:0) + C16:0]

(4) UFA = Σ(MUFA) + Σ(PUFA).

The IA measures the relationship between pro-atherogenic saturated fatty acids (which favor lipid plaque accumulation) and anti-atherogenic unsaturated fatty acids, calculated using the relationship between C12:0, C14:0, C16:0 and MUFAs/PUFAs. Concurrently, IT evaluates the tendency of the lipid matrix to form blood clots within the vascular system, contrasting thrombus-inducing saturated fats (C14:0, C16:0, C18:0) against anti-thrombogenic MUFAs, PUFAs-n6, and PUFAs-n3. Alongside these, the Health-Promoting Index (HPI) was applied as an inverse parameter of atherogenicity, where higher values mathematically reflect a superior protective effect against circulatory disease development. The calculated chronological shifts for these nutritional metrics across both ovine genotypes are compiled in Table 2.

The mathematical modeling of these indices in Table 2 revealed divergent, breed-specific cardiovascular health-promoting trajectories across the postpartum timeline. In the high-yielding Chios breed, both risk indices exhibited a highly linear, progressive decline from the raw colostrum at DPP 1 (IA = 2.384; IT = 1.435) through the final mature milk checkpoint at DPP 15 (IA = 1.638; IT = 1.077). This continuous linear drop indicates a rapid postpartum metabolic suppression of medium-chain saturated fats (C12:0, C14:0, and C16:0) that typically inhibit cellular low-density lipoprotein receptor (LDLR) clearance pathways [53]. Concurrently, the HPI values for Chios increased progressively over time, moving from an initial 7.76 at birth to a peak of 10.56 at DPP 5, reflecting an expanding profile of nutritional quality as the secretome matured.

Conversely, the indigenous Karagouniko breed displayed a specialized, non-linear buffered trajectory. Karagouniko colostrum at DPP 1 possessed an exceptionally optimized baseline atherogenic and thrombogenic profile (IA = 1.533; IT = 1.093) paired with a maximized Health-Promoting Index (HPI = 9.50). This establishes that the resilient, indigenous genotype (Karagouniko) delivers an inherently highly fluid, anti-atherogenic functional lipid mixture to the newborn lamb immediately at birth. As the Karagouniko lactation cycle entered the intensive transitional phase at DPP 9, the activation of de novo short- and medium-chain saturated lipid assembly within the mammary gland temporarily elevated these risk values (IA = 1.864; IT = 1.431; HPI = 10.10). However, these parameters successfully normalized and recovered by DPP 15 (IA = 1.480; IT = 1.205; HPI = 12.35), providing stable functional dairy matrix suitable for further nutritional evaluation and potential dietary applications.

The polyunsaturated fatty acid to saturated fatty acid ratio (PUFA/SFA)—a metric widely used to evaluate dietary fat impacts on human low-density lipoprotein cholesterol (LDL-C) and cardiovascular risk profiles—ranged from 0.07 to 0.10 in the Chios breed and 0.09 to 0.10 in the Karagouniko breed across the temporal timeline. This tight, low range aligns with classical dairy fat thresholds (0.02–0.17) noted in human dietary models [54], while these PUFA/SFA ratios consistently mirrored these shifts, establishing these ruminant fractions as high-quality functional lipid matrices.

However, because classical PUFA/SFA models fail to account for the powerful, lipid-clearing benefits of monounsaturated fatty acids (MUFAs), such as Oleic acid (C18:1 cis-9), which upregulate low-density lipoprotein receptor (LDLR) clearance pathways [53], the comprehensive tracking of IA, IT, and HPI ensures a much more accurate characterization of the nutritional and functional evolution of these secretomes.

To provide an overview comprehensive synthesis of the longitudinal biological trajectories characterizing early ovine secretome dynamics, the full set of immunochemical kinetics, bioactive protein profiles, multivariate lipidomic patterns, and cardio-protective quality indices were mapped alongside their respective two-way RM-ANOVA metrics (Table 3). Overall, this trajectory analysis demonstrates that while Chios ewes exhibit higher initial secretory throughput for key bioactives, Karagouniko ewes maintain superior metabolic stability and highly favorable nutritional lipid profiles across the early postpartum interval.

## 3. Discussion

### 3.1. Molecular and Evolutionary Mechanisms of Whey Immunological Secretions

#### 3.1.1. Temporal Clearance Trajectories and Evolutionary Genetic Diversification of Absolute IgG

The postpartum clearance kinetics of maternal Immunoglobulin G (IgG) across both Greek ovine genotypes underscore a highly coordinated, time-dependent metabolic shift within the mammary epithelium (Figure 1). A highly significant Breed Χ Time interaction (*p* < 0.0001) confirms distinct genetic regulation governing immune protein depletion. In the high-yielding Chios breed, the sharp drop from 69.29 ± 7.33 mg/mL at DPP 1 to mature baseline levels by DPP 3 reflects a specialized dilution effect driven by accelerated milk volume synthesis. Conversely, the indigenous Karagouniko breed exhibits a buffered, gradual downward trajectory from its initial maximum of 44.06 ± 2.37 mg/mL, retaining elevated IgG levels through DPP 9 (see Section 2.1.1 and Table 3).

Phenotypically, this variance reflects distinct evolutionary selective pressures. Indigenous livestock like Karagouniko evolved under natural, extensive selection where environmental stressors and pathogen exposure required sustained passive immunity to protect neonates against localized endemic pathogens under grazing conditions [55].

In contrast, artificial selection of the Chios breed for high milk yield is accompanied by distinct clearance kinetics of maternal immunoglobulins, likely reflecting differential transcellular transport dynamics within alveolar cells [56]. This results in a dense initial immunoglobulin release followed by a swift shift to high-volume nutritional secretome synthesis, highlighting how selection for yield modifies the physiological clock governing the immune-to-nutritive transition.

#### 3.1.2. Chemometric Dissection of IgG Variance: Selection Uniformity vs. Outlier Dynamics

The unsupervised Principal Component Analysis (PCA) of temporal IgG matrices visually confirms how genetic background influences the maternal immune repertoire (Figure 2). The distinct separation captured in both models—explaining 91.7% of cumulative variance in Chios (PC1 = 71.1%, PC2 = 20.6%) and 89.5% in Karagouniko (PC1 = 71.7%, PC2 = 17.8%)—demonstrates a highly organized, time-dependent molecular path. However, the orientation of the 95% confidence ellipses reveals contrasting biological dynamics.

The tight spatial clustering in Karagouniko ewes indicates a synchronized physiological pattern reflecting long-term environmental adaptation under extensive grazing conditions (Figure 2B). In contrast, the expanded ellipse of Chios ewes (Figure 2A) reveals greater individual variance during intermediate lactation. Intensive selection for milk volume can partially decouple immune transport systems from yield, causing some ewes to maintain high IgG clearance thresholds while others undergo rapid dilution of immune fractions, prioritized by a physiological high milk volume production in the improved genotype. Previously, breeding selection criteria focused primarily on production traits, including milk yield, which may have negatively affected mammary morphology by increasing udder depth and reducing teat verticality [57,58,59,60]. Although selection for production traits can negatively impact mammary morphology, traditional Greek breeds maintain lower mastitis prevalence [61]. To ensure optimal methodological rigor, cohort allocation, appropriate study size determination in alignment with STROBE Item 10 reporting principles (detailed in Supplementary Table S5), but also to account for this variance, cohort size (*n* = 10 Karagouniko vs. *n* = 5 Chios) was balanced against strict inclusion criteria—matching parity (1.6 vs. 1.9), uniform age, and low baseline SCC (<2.5 × 10^5^ cells/mL)—ensuring high genetic/health homogeneity and robust statistical power across the N = 75 longitudinal sampling events (DPP 1 -15). Practically, this highlights that Chios ewes require stricter individual screening during colostrum collection to guarantee consistent immunoglobulin yields for functional foods.

#### 3.1.3. Biomolecular Diagnostics for Milk Adulteration and Secretory Phase Verification

Beyond neonatology, non-overlapping absolute IgG concentration boundaries provide a sensitive diagnostic tool for dairy food safety and authentication. Fraudulent or accidental inclusion of early transitional milk or late colostrum into standard milk supplies destabilizes thermal pasteurization and alters sensory profiles in high-value products like Protected Designation of Origin (PDO) cheeses [62].

Because standard physicochemical parameters (total protein, fat, density) overlap significantly between transitional fluids (pre-mature) and mature milk, they lack diagnostic precision. Absolute IgG kinetics establish clear operational baselines: values exceeding the critical 2.0 mg/mL threshold serve as a specific biomarker for early transitional fluid (DPP 1 -5). Integrating these quantitative thresholds into automated ELISA or microfluidic biosensing platforms enables high-throughput verification of raw sheep milk authenticity prior to industrial processing [63,64,65,66,67,68,69].

#### 3.1.4. Secretory Whey Lactoferrin Kinetics and Neonatal Mucosal Fortification

Secretory whey Lactoferrin (Lf) displayed parallel, breed-independent depletion kinetics characterized by a non-significant Breed X Time interaction (*p* = 0.5877) and a dominant Time effect (71.56% of variance). The synchronized drop from peak levels at DPP 1 (467.5 μg/mL in Chios; 402.1 μg/mL in Karagouniko) to a steady-state plateau from DPP 5 onward indicates that Lf expression operates independently of selection pressures governing milk yield or IgG clearance (Figure 4) (see Section 2.1.1 and Section Synchronized, Breed-Independent Clearance Kinetics and Table 3). It should be noted as a methodological limitation that Lf was identified via electrophoretic mobility (~78–80 kDa) and standardized molecular weight estimation rather than direct immunochemical validation (e.g., Western blot or LC-MS/MS); future studies will incorporate specific antibody-based assays to further confirm absolute specificity. However, the molecular weight estimation derived from colostrum whey fraction, is in agreement and supported by previous reports [16,18,33].

This structural conservation underscores Lactoferrin’s essential role as a non-specific mucosal barrier against neonatal pathogen colonization [70]. From a mechanistic standpoint, secretory Lactoferrin exerts a high-affinity iron-sequestration effect. This binds free ferric (Fe^3+^) ions in the intestinal lumen, starving iron-dependent enteric pathogens such as *Escherichia coli*, *Salmonella enterica* and paratuberculosis in many animals including ruminants caused by *Mycobacterium avium* spp. *paratuberculosis* [71], while selectively fostering the expansion of beneficial, iron-independent probiotic microflora like *Bifidobacterium* species [72].

Furthermore, the retention of genotype-invariant equilibrium concentrations of lactoferrin through DPP 15 confirms its ongoing role in supporting intestinal epithelial differentiation, fortifying tight junctions, and dampening pro-inflammatory cytokine pathways (IL-1*β*, IL-6, TNF-*α*) via competitive down-regulation of NF *κ*B activation [73].

### 3.2. Transitional Lipidomics and Bioactive Saturated and Unsaturated Fatty Acid Flux

Gas chromatography analysis of fatty acid methyl esters (FAME) reveals major lipid remodeling during the colostrum-to-milk transition. Reductions in long-chain saturated fatty acids (LC-SFAs; e.g., palmitic acid C16:0) alongside shifting short-chain (SCFAs; C4:0–C8:0) and medium-chain fatty acids (MCFAs; C10:0–C12:0) reflect a coordinated, breed-specific metabolic shift. Pre-partum mammary epithelium relies on taking up preformed circulating fatty acids mobilized from adipose tissue or diet. Postpartum, the accumulation of volatile SCFAs and MCFAs is consistent with the classic upregulation of de novo lipogenic pathways traditionally governed by acetyl-CoA carboxylase (ACC) and fatty acid synthase (FAS) [51,74,75].

On PCA biplots, Karagouniko ewes follow a steady, sequential metabolic progression originating from colostrum rich in oleic (C18:1) and palmitic (C16:0) acids. Conversely, Chios ewes exhibit a non-linear parabolic trajectory driven by intense de novo SCFA and MCFA assembly during intermediate lactation (DPP 3 -5) (see Section 2.3.1, Table 1 and Table 3). This post-partum surge of C4:0–C10:0 provides rapidly oxidizable fuel to meet early physiological demands in high-yielding dairy genotypes prior to mature milk stabilization [76,77].

#### Interpretation of PCA Biplot Trajectories: Linear vs. Parabolic Lipogenic Programming

Multidimensional positioning of fatty acid loading vectors relative to sample scores illustrates divergent metabolic switch-over mechanisms between breeds. In Karagouniko ewes, directional movement along PC1 reflects a gradual transition. Early colostrum is characterized by long-chain structural fats (C18:0, LC-SFAs) mobilized from maternal reserves. As lactation advances, strong loading of MUFA, PUFA, and CLA vectors toward DPP 9 and DPP 15 space indicates a systematic linear shift toward mature milk fat synthesis, maintaining oleic acid (C18:1) as the predominant fatty acid throughout (see Section 2.3.1 and Table 3). [42,78]. This stable reliance on systemic lipid mobilization optimizes the fluid behavior of the milk matrix without demanding drastic, abrupt transitions in local epithelial machinery.

Crucially, the maintenance of oleic acid (*C*18:1) as the predominant fatty acid for the Karagouniko breed throughout the 15-day timeline confirms a highly stable, continuous reliance on peripheral fat mobilization to support lactation without demanding major abrupt surges in local synthesis.

Conversely, the parabolic trajectory of Chios ewes demonstrates a biphasic adjustment. Migration of DPP 3 and DPP 5 samples to positive PC1 space (PC1 = +2.71 and +2.57) aligns with SCFA/MCFA vectors (C4:0–C12:0), reflecting an intense temporal shift toward short- and medium-chain fatty acid accumulation immediately following colostrum clearance, indicative of localized de novo lipogenic prioritization. By DPP 15, this surge subsides (PC1 = −1.72), re-aligning with mature milk fat components, structural fats, and unsaturated lipids [51,79]. Thus, while Karagouniko ewes undergo gradual lipid reprogramming heavily anchored by systemic fat mobilization, Chios ewes prioritize an immediate, intense surge in short- and medium-chain fatty acid synthesis, likely driven by differential expression of key lipogenic enzymes or varying energy-balance sensitivities postpartum [51].

### 3.3. Computational Modeling of Nutritional Quality and Cardiovascular Risk Indices

#### 3.3.1. Analytical Limitations of Classical PUFA/SFA Predictive Ratios

Evaluating the secretome using the standard polyunsaturated to saturated fatty acid ratio (PUFA/SFA) yielded a narrow range (0.07–0.10) across all timepoints for both breeds. Although consistent with baseline ruminant thresholds (0.02–0.17), this simple ratio fails to capture modern cardiovascular risks because it ignores the protective effects of SFAs on HDL-cholesterol [54,80].

The primary limitation of PUFA/SFA is its inability to distinguish individual fatty acid structures within saturated or unsaturated classes. It treats all SFAs as equally harmful—ignoring the neutral behavior of stearic acid (C18:0)—and completely overlooks the cardioprotective benefits of monounsaturated fatty acids (MUFAs) like oleic acid (C18:1 cis-9). In clinical trials, oleic acid lowers plasma LDL-C by upregulating hepatic LDL receptor (LDLR) clearance [53]. Consequently, relying solely on PUFA/SFA obscures unique nutritional attributes in complex functional lipids.

#### 3.3.2. Structural Analysis of Atherogenic, Thrombogenic, and Health-Promoting Equations

To overcome these limitations, the Index of Atherogenicity (IA) and Index of Thrombogenicity (IT) proposed by Ulbricht and Southgate (1991) [52] were evaluated alongside the inverse Health-Promoting Index (HPI) developed by Chen et al. (2020) [81]. The mathematical structure of the IA equation provides a more reliable predictive model for human clinical health and superior clinical risk prediction because it explicitly targets pro-atherogenic medium-chain SFAs—lauric (C12:0), myristic (C14:0), and palmitic (C16:0)—which accelerate coronary artery disease by driving endothelial plaque deposition [82,83].

Concurrently, IT evaluates vascular thrombus risk by balancing pro-thrombotic SFAs (C14:0, C16:0, C18:0) against anti-thrombotic n−3/n−6 PUFAs, MUFAs, and CLA vectors [52]. In parallel, HPI serves as a direct indicator of lipid matrix quality, where higher values correlate with cardiovascular protection [84,85,86].

Despite their utility, these models have minor limitations. In IT, stearic acid (C18:0) remains categorized as pro-thrombotic based on legacy data, despite evidence demonstrating its neutral impact on plasma LDL-C. Additionally, IA and IT treat all n−6 PUFAs equally, oversimplifying distinct metabolic pathways (arachidonic vs. linoleic acid) and omitting trans-fatty acid isomers as reported by Ulbricht and Southgate [52]. Similarly, the HPI carries identical mathematical constraints because it operates as the direct mathematical inverse of the IA formula, highlighting the potential for future model refinements using updated clinical coefficients [81,87].

#### 3.3.3. Clinical Translation to Human Nutrition and Functional Supplement Design

Evaluating dietary lipid components through global baseline metrics is essential to confirm their commercial and clinical value for human functional food systems. Meta-analyses across agricultural products show species-specific biological programming driving atherogenic variation: IA values range from 0.084–0.55 in crops, 0.21–1.41 in fish, and 0.165–1.32 in meats, whereas conventional bovine and caprine dairy express higher risks (1.42–5.13) [54,88,89].

In this study, maximum IA values for Chios (2.384) and Karagouniko (1.864) remained at the lowest, most favorable end of the classical dairy spectrum, even during peak postpartum de novo lipogenesis. (see Table 2 and Table 3). In the indigenous Karagouniko breed, the progressive, sequential reduction in both atherogenic and thrombogenic risk metrics from DPP 1 (IA = 1.533; IT = 1.093) combined with a highly maximized Health-Promoting Index (HPI = 9.50) to DPP 15 (IA = 1.480; IT = 1.205) with an exceptional HPI = 12.35, demonstrate a smooth transition that rapidly lowers plaque-promoting SFAs and inhibit cellular low-density lipoprotein receptor activity [53].

The low atherogenic and high HPIs at maturity demonstrate that this ovine secretome yields a bioaccessible functional dairy matrix. Chios ewes display an optimized baseline at birth (IA = 2.384; IT = 1.435; HPI = 7.76), confirming that native maternal genotypes generate a cardio-protective lipid environment at lactation onset. Although intermediate de novo lipogenesis temporarily elevates risk metrics (peaking at DPP 9 in Karagouniko with IA = 1.864; IT = 1.431; HPI = 10.1), both breeds stabilize into highly safe ranges by DPP 15. Because consumption of low-IA, high-HPI dairy matrices reduces total serum cholesterol and LDL-C [90], these findings indicate that early ovine milk fractions warrant further study regarding their long-term nutritional profile.for potential health and dietary application.

## 4. Materials and Methods

**Figure 8:**
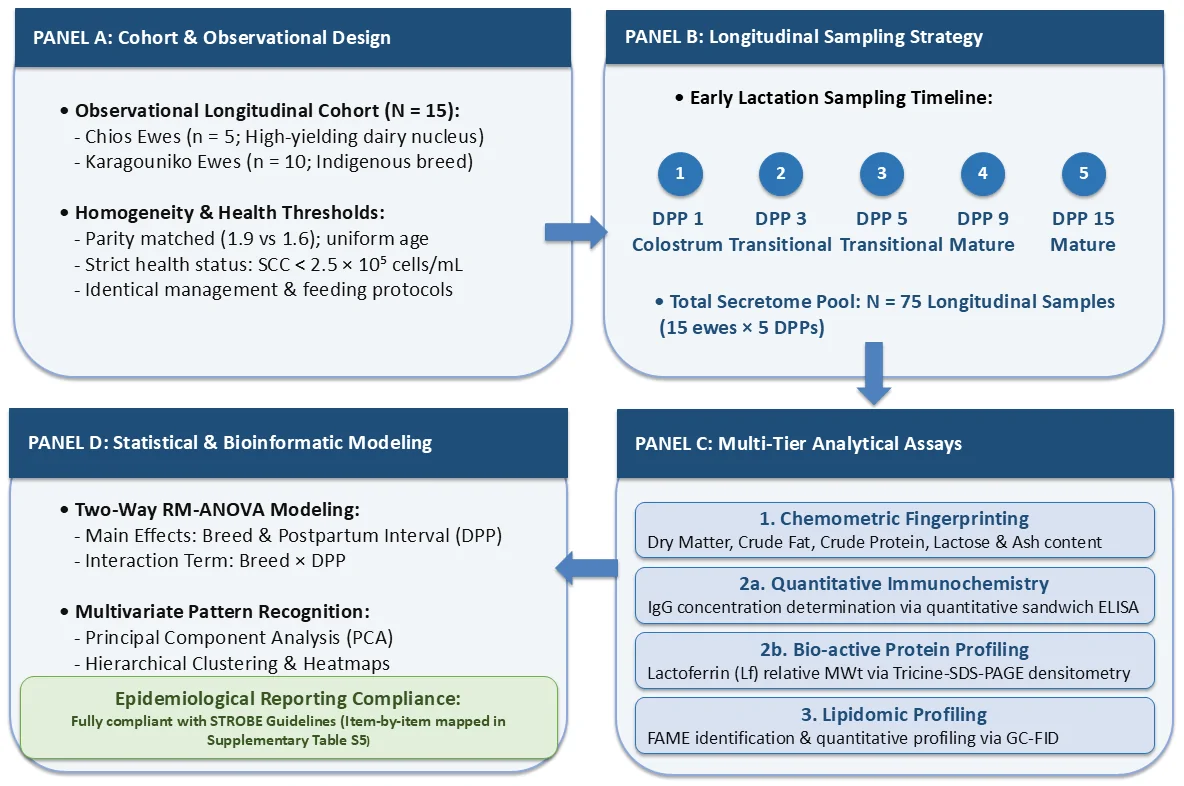
Longitudinal Experimental Architecture & Analytical Pipeline. Schematic workflow diagram illustrating the longitudinal experimental design and analytical framework of the study. Panel (**A**) Stratification and health filtering of the observational ewe cohort (*n* = 5 Chios vs. *n* = 10 Karagouniko ewes). Panel (**B**) Longitudinal colostrum and milk sampling across five postpartum intervals (DPP 1, 3, 5, 9, 15; total N = 75 observations). Panel (**C**) Parallel multi-tier analytical assays, including chemometric composition, immunochemical quantification of IgG via ELISA, densitometric semi-quantification of Lactoferrin (Lf) via Tricine-SDS-PAGE, and FAME lipidomic profiling via GC-FID. Panel (**D**) Downstream two-way repeated-measures statistical modeling and multivariate data integration.

To evaluate the longitudinal variations in colostrum and milk composition between Chios and Karagouniko ewes, a multitier analytical workflow was implemented spanning animal selection, sampling, laboratory analysis, and statistical modeling. An overview of the entire experimental architecture and analytical pipeline is schematically presented in Figure 8.

### 4.1. Animal Management, Feeding, and Sample Collection

The experimental cohort comprised fifteen ewes representing two distinct ovine genotypes native to Greece: a high-yielding dairy breed (Chios, *n* = 5) and a resilient, indigenous dual-purpose breed (Karagouniko, *n* = 10). To eliminate confounding environmental, seasonal, and nutritional variables, all experimental ewes were selected from the same production year, were of uniform age, and exhibited no significant phenotypic variation regarding baseline body weight. The mean parity of the selected cohorts was 1.9 for the Chios breed and 1.6 for the Karagouniko breed, representing homogenous physiological maturity across both experimental groups. Strict inclusion criteria were enforced prior to enrollment: all ewes underwent thorough veterinary clinical examination to verify excellent systemic health, normal body temperature, undamaged udder conformation, and the complete absence of subclinical mastitis (confirmed by Veterinarian’s inspection, mastitis test and somatic cell counts <2.5 × 10^5^ cells/mL). Lambing was naturally synchronized to ensure uniform seasonal conditions, and any animal requiring antibiotic, anti-inflammatory, or medical intervention prior to or during the 15-day study period was excluded. All animals were maintained under identical environmental conditions at the experimental farm of the Agricultural University of Athens. Throughout the study, the ewes were housed in individual pens and fed a standardized ration twice daily at 07:00 and 16:00 h. The dietary regimen consisted of high-quality alfalfa hay (energy content: 4.14 MJ/kg) supplemented with a controlled concentrate diet partitioned into a basal ration (7.1 MJ/kg) and a lactation ration (7.3 MJ/kg) tailored to meet matching physiological requirements.

Serial mammary gland colostrum and transitioning milk samples were systematically collected from each animal across a 15-day longitudinal postpartum timeline, with sampling intervals fixed at days 1 (raw colostrum), 3, 5, 9, and 15 post-partum (DPP). At each designated timepoint, approximately 300 mL of secretion was manually stripped per animal. Immediately following collection, each individual sample was thoroughly homogenized and partitioned into two distinct analytical working aliquots before being flash-frozen and stored at −20 °C until further analysis. The first aliquot was subsequently lyophilized and reserved for total lipid extraction and direct fatty acid methyl ester (FAME) composition analysis. The companion fluid aliquot was utilized for absolute protein determination, including total immunoglobulin G (IgG) quantification via ELISA and full protein profiling (including lactoferrin) via discontinuous polyacrylamide electrophoresis.

### 4.2. Protein Fractionation, Electrophoretic Analysis, and Lactoferrin Determination

#### 4.2.1. Sodium Dodecyl Sulphate Polyacrylamide Gel Electrophoresis (SDS–PAGE)

Lyophilized mammary secretion samples were reconstituted in deionized water (1:10, *w*/*v*) and centrifuged at 3000 rpm for 30 s at 4 °C to remove residual fat. Caseins were subsequently precipitated by adjusting the mixture to pH 4.6 via the dropwise addition of 1 M acetic acid (cat No. 137130, Merck, Darmstadt, Germany) under continuous stirring. The acidified homogenate was centrifuged at 3000 rpm for 10 min at 4–15 °C to separate the whey supernatant from the raw casein pellet. The resulting casein pellet was washed three times with deionized water and re-centrifuged under identical conditions. Both isolated fractions were stored at 4 °C prior to downstream biochemical analysis.

For electrophoretic separation, whey aliquots (3 μL) were denatured by mixing with 1 μL of 2-mercaptoethanol (cat no. M6250, Sigma-Aldrich, St. Louis, MO, USA), 12.5 μL of lithium dodecyl sulfate (LDS) sample buffer (cat no. NP0009, Thermo Fisher Scientific, Waltham, MA, USA), and 8.5 μL of deionized water. For comparative profiling, casein samples (40 mg) were solubilized in 12.5 μL of 2 M NaOH (cat no. 106498, Merck, Darmstadt, Germany) before being supplemented with 1 μL of 2-mercaptoethanol and 6 μL of LDS sample buffer. All prepared samples were denatured thermally at 85 °C for 3 min, rapidly quenched on ice, and concentrated via centrifugation (9000 rpm for 30 s). A prestained molecular weight standard ladder (Thermo Fisher Scientific, Waltham, MA, USA) was routinely included in each individual run to map the migration boundaries. As positive Lf control was used Bovine lactoferrin, (cat no. LTF-229B Creative BioMart, Shirley, NY, USA).

High-resolution discontinuous Tricine–SDS–PAGE was executed to achieve clean resolution of the high-molecular-weight whey proteins. Resolving and stacking gel matrices were prepared utilizing an acrylamide/bis-acrylamide (29:1) stock solution (cat no. 1610156, Bio-Rad, Hercules, CA, USA) buffered with Tris–HCl at pH 8.8 and pH 6.8, respectively, and polymerized using sodium dodecyl sulfate (SDS) (cat no. 110732, Sigma-Aldrich, St. Louis, MO, USA), ammonium persulfate (APS) (cat no. A3678, Sigma-Aldrich, St. Louis, MO, USA), and tetramethylethylenediamine (TEMED) (cat no. 110732, Sigma-Aldrich, St. Louis, MO, USA). Electrophoretic separation was conducted using a vertical tank system (18 × 24 cm plates, Code No. 80-6189-82, Model SE660, Pharmacia Biotech, Uppsala, Sweden) maintained at a constant voltage of 190 V for 4–5 h at a controlled temperature of 4 °C. Following running completion, gels were fixed and stained with Coomassie Brilliant Blue R-250 (cat no. 1.12553, Sigma-Aldrich, St. Louis, MO, USA) for 30 min, and subsequently cleared in an aqueous methanol–acetic acid destaining solution until stable, high-contrast visualization of distinct protein bands was achieved. Target ovine lactoferrin (Lf) was identified specifically within the whey lanes based on its characteristic electrophoretic mobility and migration profile at approximately 78–80 kDa. which was in agreement with previous reports [16,18,33]. Future studies will incorporate specific antibody-based assays to further confirm band size and specificity.

#### 4.2.2. Protein Quantification and Lactoferrin Determination

For absolute quantitative chemometrics, the destained polyacrylamide matrices were positioned on a stabilized white-light transilluminator box. High-resolution digital captures were acquired using a Nikon D3500 digital single-lens reflex (DSLR) camera (Nikon Corporation, Tokyo, Japan) equipped with a standardized focal lens. To eliminate electronic exposure artifacts, all manual camera capture parameters—including aperture, shutter speed, ISO sensitivity, and white balance—were locked and kept strictly constant across all gel profiles. Images were saved and exported in uncompressed, tagged image file format (TIFF). Digital densitometric profiling and boundary lane integrations were performed using ImageJ software (v1.54, National Institutes of Health, Bethesda, MD, USA) [91]. Absolute mass quantification of target lactoferrin bands was determined by calculating their integrated optical density (IOD) relative to a parallel internal standard curve generated by a purified reference standard used as loading control (Section 4.2.1, Bovine lactoferrin ~77.9 kDa) of known concentration loaded onto the identical gel matrix to calculate absolute yield profiles. Final values were mathematically converted and expressed as micrograms of lactoferrin per milliliter of whey (μg/mL).

Considering that in our study Lf profiling by densitometry of protein bands on SDS-PAGE gels stained with Coomassie is semi-quantitative evaluation the implementation of commercial ELISA is worth-mentioning as a recommended next step approach with antibody-based assays for absolute biochemical benchmarking.

### 4.3. Immunoglobulin G (IgG) Determination

The concentration of immunoglobulin G (IgG) in ovine colostrum samples was determined via a sandwich Enzyme-Linked Immunosorbent Assay (ELISA), the Calokit Ovino test kit (CALOKIT-OVINO, ZEU-IMMUNOTEC/ZeuLab, Zaragoza, Spain; catalogue no. ZE/CALOKIT-OVINO, order code ZE/CKO96), following the standard running protocol provided by the manufacturer.

Briefly, colostrum samples were thawed at 4 °C for 24 h prior to analysis. Depending on the postpartum day, samples were diluted with deionized water (1:10, 1:20, 1:100, or 1:200), centrifuged at 5000 rpm for 10 min to separate the serum fraction, and subsequently diluted to a final ratio of 1:2000 before analysis. IgG quantification was performed using standard solutions ranging from 0.00 to 0.75 mg/mL. A second-order polynomial standard curve was generated by plotting optical density (OD) values against known concentration increments, and sample targets were explicitly resolved via mathematical interpolation from the resulting calibration curve.

### 4.4. Determination of Fatty Acid Composition

Fatty acids composition was established by direct transesterification and methylation of lyophilized samples as previously described by Massouras et al. [92].

Briefly, a quantity of 150 mg of lyophilized colostrum was directly methylated with 2 mL of 0.5 M sodium methylate (cat no. 818194, Merck, Darmstadt, Germany), followed by the addition of 2 mL of 140 g/L boron trifluoride (cat no. B1252, Sigma-Aldrich, St. Louis, MO, USA) in methanol (BF3) at 50 °C for 30 min. The isolated fatty acid methyl esters (FAMEs) were separated and quantified utilizing a Shimadzu gas chromatograph (model GC-17A, Kyoto, Japan) equipped with a high-sensitivity flame ionization detector (FID).

A highly polar SP-2560 fused-silica capillary column (60 m × 0.25 mm I.D., 0.20 µm; film thickness; Supelco, Bellefonte, PA, USA) was employed for FAME separation. The injector split ratio was dialed to 1:50 and helium was maintained as the carrier gas at a constant column flow rate at 1.8 mL/min. Operating temperatures for the injection port and detector block were 250 °C and 270 °C, respectively, using an injection volume of 1 μL.

Individual fatty acids were identified via direct comparisons of peak retention times and integration areas against a certified commercial reference standard mix (Supelco 37 Component FAME Mix, Sigma-Aldrich; Merck KGaA, Darmstadt, Germany). Chromatographic peak integrations were handled using GC Solution software (version 2.30, Shimadzu Corporation, Kyoto, Japan). Individual fatty acid (FA) concentrations were expressed as a relative weight percentage (% *w*/*w*) of total detected FAMEs. Distinct nutritional lipid classes were calculated according to the following grouping equations:

Saturated fatty acids (SFA) = C4:0 + C6:0 + C8:0 + C10:0 + C12:0 + C14:0 + C16:0 + C18:0 + C20:0; Monounsaturated fatty acids (MUFA) = C14:1 + C16:1 n−7 + C18:1 n−9; Polyunsaturated fatty acids (PUFA) = CLA + C18:2 n−6 + C18:3 n−3.

### 4.5. Statistical Analysis

#### 4.5.1. Two-Way Mixed-Factor Repeated-Measures ANOVA of Bioactive Components

A two-way repeated-measures analysis of variance (RM ANOVA) was employed to model the longitudinal kinetics of the concentrations of Lactoferrin (Lf) and immunoglobulin G (IgG) in the samples of the Karagouniko and Chios breeds across the early lactation timeline.

The statistical framework integrated one between-subjects factor (Breed: Karagouniko vs. Chios) and one within-subjects repeated factor (Time: 1, 3, 5, 9, and 15 DPP) with matching by columns to rigorously assess main treatment effects and evaluate corresponding cross-interaction vectors (Breed X Time). Normality of data distribution across all variables and residual homoscedasticity were verified using the Shapiro–Wilk test as optimal for small-to-moderate sample sizes (*n* = 5–10), prior to testing. Sphericity of the variance-covariance matrix was evaluated using Mauchly’s test, where the assumption of sphericity was violated (*p* < 0.05), degrees of freedom and *p*-values were adjusted using Greenhouse-Geisser corrections. The proportion of total variance explained by model main factors and interaction terms was derived directly from the two-way ANOVA sum of squares decomposition (SSFactor/SSTotal and partial eta-squared, *η*p2).

Following the detection of a statistically significant main effect or interaction, post-hoc pairwise multiple comparisons were performed using Bonferroni’s adjustment to control the family-wise error rate.

To track postpartum protein depletion trends, Day 1 post-partum (colostrum) was designated as the baseline reference control column against which all subsequent production timepoints (Days 3, 5, 9, and 15 DPP) were compared. Statistical significance was defined a priori at *p* < 0.05. On all generated graphical displays, significance brackets and localized asterisks depict adjusted post-hoc tests (* *p* < 0.05, ** *p* < 0.01, *** *p* < 0.001, **** *p* < 0.0001; ns = non-significant). All hypothesis testing calculations, variance splits, and curve fittings were implemented utilizing GraphPad Prism software (v5.04; GraphPad Software, San Diego, CA, USA).

#### 4.5.2. Data Pre-Processing and Normalization

Prior to multivariate analysis, data cleaning and breed-specific median imputation were performed using the pandas (v2.1.1) and scikit-learn (v1.3.1) libraries for Python (Python v3.11.5, 64-bit) [93,94]. To maintain the biological integrity of the longitudinal profile, missing IgG (or Lactoferrin) values were replaced with the median concentration of the respective breed at that specific time point. To account for the large differences in magnitude between early colostrum (Day 1) and late transitional milk (Day 15), data were standardized to unit variance (mean = 0, standard deviation = 1) using a standard scaling transformation.

#### 4.5.3. Dimensionality Reduction (PCA) and Multivariate Data Visualization

Multivariate relationships between breed origins, transitional timelines, and overall protein-lipid secretion trends were visualized via Principal Component Analysis (PCA) utilizing either the online SRPlot biological data visualization platform [95] or as described earlier in the results Section 2.1.2. The analysis utilized the singular value decomposition (SVD) method to reduce the dimensionality of the longitudinal time points into two primary coordinates (PC1 and PC2). The resulting unsupervised score plots were annotated with 95% confidence ellipses to statistically delineate breed-specific clustering (Karagouniko vs. Chios). Variable loading trajectories (rotation vectors) were calculated and superimposed to determine the relative contribution and directional correlation of each specific time point (DPP 1 through DPP 15) to the total observed biological variance.

#### 4.5.4. High-Dimensional Advanced Data Visualization

Comprehensive multivariate data plots, matrix heatmaps, and side-by-side comparative breed biplots were generated to facilitate publication-quality visual synthesis. Complex data visualization configurations, including vector overlays, multi-panel coordination, and high-dimensional plotting were developed using custom Python-based scientific plotting libraries (including Matplotlib v3.8 and Seaborn v0.13) to evaluate breed-specific clustering and temporal effect on the fatty acid compositions Principal Component Analysis (PCA). Alongside, SRPlot web-based data visualization online platform was employed to evaluate breed-specific clustering and temporal IgG PCA (Science Research Plot), https://www.srplot.net/ (assessed on 3 July 2026). The analysis utilized the singular value decomposition (SVD) method to compute principal components. The resulting score plots were annotated with 95% confidence ellipses to delineate breed-specific clustering (Karagouniko vs. Chios). Variable loading trajectories were included to determine the contribution of each time point (DPP 1 through DPP 15) to the total observed variance.

#### 4.5.5. STROBE Checklist: Reporting Guidelines

To ensure transparent and comprehensive reporting of our observational longitudinal design, this study adheres to the Strengthening the Reporting of Observational Studies in Epidemiology (STROBE) guidelines, with item-by-item location mappings detailed in Supplementary Table S5.

## 5. Conclusions

The systematic monitoring of the early post-partum ovine secretome across Chios and Karagouniko ewes reveals a highly synchronized biological transition with strong applications for functional food design and targeted human nutrition. By combining multivariate chemometrics with quantitative immuno-lipidomic profiling, this study successfully mapped the temporal trajectories of key immunological and fatty acid components during the colostrum-to-milk transition (DPP 1 to DPP 15). Our findings demonstrate that while maternal immune protein clearance and mammary lipid pathway remodeling follow a unified biological timeline, the structural kinetics of these processes are strongly governed by breed-specific genetics. Specifically, the unselected Karagouniko genotype maintains a buffered clearance slope for systemic IgG compared to the rapid dilution observed in the high-yielding Chios breed, whereas mucosal lactoferrin exhibits a conserved, breed-independent depletion trajectory. Crucially, these non-overlapping concentration boundaries establish a high-accuracy baseline to verify raw post-partum secretory phases, protecting commercial dairy channels against colostrum contamination.

Concurrently, the transitional lipidomics data document a major biochemical shift from adipose tissue mobilization toward localized de novo fatty acid synthesis. Evaluating these secretomes through clinical lipid indices (IA, IT, and HPI) confirms a favorable nutritional profile, with both genotypes stabilizing into balanced, low-atherogenic profiles by DPP 15. From a biotechnological perspective, these distinct concentration windows outline optimal post-partum timepoints for harvesting high-purity immunoglobulins, bioactive peptides, and cardio-protective lipid fractions tailored for specialized health supplements and immune-modulating functional foods.

Envisaging these phenotypic findings could provide a necessary baseline for future molecular research. Subsequent investigations should focus on functional gene expression assays and expression quantitative trait loci (eQTL) mapping to pinpoint the regulatory networks driving these breed-specific lipogenic approaches. Furthermore, as advanced gene editing technologies continue to evolve in livestock biotechnology, integrating these genomic methods could help decouple production traits from immunological substitutions, provided such innovations are guided by rigorous bioethical oversight. Ultimately, preserving the adaptive metabolic programming of indigenous livestock remains essential for sustainable animal breeding and precision nutrition.

## Figures and Tables

**Table 1:** Fatty acid composition (%) of Chios and Karagouniko colostrum during colostrum–to-mature milk transition.

					Time (DPP)					
	1		3		5		9		15	
Fatty Acids During Lactation	C	K	C	K	C	K	C	K	C	K
***C4:0***	3.66	3.30	3.53	4.03	4.67	5.16	3.65	3.73	3.48	3.78
***C6:0***	1.18	1.46	1.90	2.78	1.85	2.30	1.85	2.16	1.59	2.00
***C8:0***	1.67	1.41	1.92	2.84	1.98	2.86	1.80	2.54	1.43	1.78
***C10:0***	4.29	3.45	5.07	6.94	5.10	6.46	4.67	6.34	3.34	3.84
***C12:0***	3.30	2.72	3.08	3.65	3.00	3.44	2.76	3.29	2.67	2.60
***C14:0***	12.90	9.60	9.81	8.62	8.32	8.27	8.63	9.56	9.08	8.32
***C16:0***	30.74	26.99	28.47	25.79	26.28	25.45	26.69	27.17	26.39	26.56
***C16:1 trans***	1.81	1.77	1.26	1.09	1.02	1.27	1.30	1.20	1.48	1.58
***C16:1 cis***	0.84	0.66	0.59	0.53	0.65	0.67	0.59	0.58	0.57	0.63
***C17:0***	0.28	0.55	0.26	0.28	0.20	0.34	0.37	0.21	0.40	0.28
***C18:0***	5.81	6.63	7.39	8.37	9.10	10.02	8.12	8.68	7.50	10.59
***C18:1***	28.56	36.24	30.63	29.31	32.27	30.67	31.09	30.64	32.17	34.42
***C18:2 n−6***	3.54	3.57	2.86	3.71	4.09	3.49	2.85	3.73	3.48	4.35
***C18:3 n−3***	0.62	1.32	1.37	0.76	0.43	0.55	2.23	0.52	1.16	0.49
***CLA***	0.55	0.89	0.76	1.09	0.78	0.55	0.88	0.18	1.05	0.70
***SFA***	63.83	56.11	61.43	63.30	60.50	64.30	58.54	63.68	55.89	59.75
***MUFA***	31.22	38.67	32.47	30.93	33.94	32.61	32.98	32.42	34.21	36.63
***PUFA***	4.71	5.78	4.99	5.56	5.30	4.58	5.95	4.43	5.70	5.54
***MC SFA***	7.14	6.31	8.9	12.56	8.93	11.62	8.32	11.04	6.37	7.63
***LC SFA***	14.03	10.82	10.65	9.43	9.17	9.28	9.59	10.35	10.04	9.23

Notes: C, Chios breed; K, Karagouniko breed.

**Table 2:** Fatty acid quality profiles and lipidomic indices of colostrum in Chios and Karagouniko sheep breeds across days post-partum (DPP).

Days Post Partum (DPP)	Atherogenicity Index (LA)		Thrombogenicity Index (IT)		Health Promoting Index (HPI)		PUFA/SFA Ratio	
	Chios	Karagouniko	Chios	Karagouniko	Chios	Karagouniko	Chios	Karagouniko
1	2.384	1.533	1.435	1.093	7.76	9.5	0.07	0.1
3	1.889	1.751	1.261	1.225	9	10.04	0.08	0.09
5	1.594	1.667	1.145	1.353	10.56	12.05	0.09	0.07
9	1.643	1.864	1.078	1.431	10.18	10.1	0.1	0.07
15	1.638	1.48	1.077	1.205	9.67	12.35	0.1	0.09

**Table 3:** Findings Overview: Comparative Ovine Secretome Analysis and Temporal Trajectories of Bioactive Proteolipidomic Dynamics.

Parameter/Category	High-Yielding Breed (Chios)	Resilient Native Breed (Karagouniko)	Statistical Metric
**Cohort Size (n)**	*n* = 5	*n* = 10	Parity & Age matched
**Peak Total IgG (DPP 1)**	69.29 ± 7.33 mg/mL	44.06 ± 2.37 mg/mL	*p* < 0.001
**IgG Clearance Kinetics**	Rapid, steep initial decline by DPP 3 (12.8 mg/mL) due to accelerated milk volume dilution.	Gradual, buffered depletion slope maintained through DPP 9.	Clear divergence in clearance rate
**IgG RM-ANOVA Metrics**	—	—	Significant Breed X Time Interaction: F(4, 52) = 14.87, *p* < 0.0001 (4.53% variance). Main Effect of Time: *p* < 0.0001 (89.14% variance).
**Peak Lactoferrin (DPP 1)**	467.5 μg/mL	402.1 μg/mL	*p* < 0.05
**Lactoferrin Clearance Kinetics**	Parallel, synchronized, breed-independent biphasic drop.	Rapid early decline by DPP 3 reaching a low-concentration steady-state plateau by DPP 5-15.	Synchronized clearance across breeds
**Lactoferrin RM-ANOVA Metrics**	—	—	Non-Significant Breed X Time Interaction: F(4, 24) = 0.72, *p* = 0.5877 (2.63% variance). Governed Overwhelmingly by Time: *p* < 0.0001 (71.56% variance).
**Fatty Acid Dynamics & Lipogenesis Mode**	Parabolic PCA Trajectory: Early post-partum surge (DPP 3-5) in short-/medium-chain FAs (C4:0–C10:0) driven by localized de novo lipogenesis in mammary epithelial cells.	Sequential/Linear PCA Trajectory: Sustained reliance on systemic adipose tissue mobilization. Oleic acid (C18:1) remains the dominant FA throughout (DPP 1-15).	Distinct metabolic/lipogenic pathways
Index of Atherogenicity (IA) * Trend	Linear, progressive suppression from DPP 1 (2.384) to DPP 15 (1.638).	Non-linear/buffered baseline starting at 1.533 (DPP 1), peaking at 1.864 (DPP 9), and normalizing to 1.480 (DPP 15).	Favorable overall trajectory
Index of Thrombogenicity (IT) * Trend	Steady reduction from DPP 1 (1.435) to DPP 15 (1.077).	Starts low at DPP 1 (1.093), transiently rises at DPP 9 (1.431), and recovers at DPP 15 (1.205).	Temporal cardio-protective profiling
Health-Promoting Index (HPI) * Trend	Rises from 7.76 (DPP 1) to a peak of 10.56 (DPP 5), stabilizing at 9.67 (DPP 15).	Begins at 9.50 (DPP 1), reaching a maximum of 12.35 by DPP 15.	Superior native breed stability
**Diagnostic & Functional Application**	IgG threshold > 2.0 mg/mL acts as a biomarker to detect early transitional fluid and prevent late-colostrum adulteration in commercial milk.	Favorable lipid indices validate use for targeted nutraceuticals and human cardio-protective functional foods.	Industry & clinical translation

Notes: DPP, Days Post-Partum; FA, Fatty Acids; (*), See details in Section 2.3.2.

## Data Availability

The original contributions presented in this study are included in the article/Supplementary Material. Further inquiries can be directed to the corresponding author.

## Supplementary Materials

The following supporting information can be downloaded at: https://www.mdpi.com/article/10.3390/ijms27167252/s1.

## Abbreviations

The following abbreviations are used in this manuscript:

ANOVA	Analysis of Variance
DPP	Days Post-Partum
ELISA	Enzyme-Linked Immunosorbent Assay
FA	Fatty Acid
GC	Gas Chromatography
HPI	Health-Promoting Index
IA	Atherogenicity Index
IgG	Immunoglobulin G
IT	Thrombogenicity Index
Lf	Lactoferrin
MUFA	Monounsaturated Fatty Acid
PCA	Principal Component Analysis
PUFA	Polyunsaturated Fatty Acid
SFA	Saturated Fatty Acid
Tricine–SDS–PAGE	Tricine–Sodium Dodecyl Sulfate–Polyacrylamide Gel Electrophoresis

## References

[1] Ahmadi F., Kim S., Hwangbo D., Oh Y., Yu J., Bae J., Kim N.Y. (2021). Performance of Hanwoo calves fed a commercial colostrum replacer versus natural bovine colostrum. J. Anim. Sci. Technol..

[2] Baumrucker C.R., Gross J.J., Bruckmaier R.M. (2023). The importance of colostrum in maternal care and its formation in mammalian species. Anim. Front..

[3] Poonia A., Shiva (2022). Bioactive compounds, nutritional profile and health benefits of colostrum: A review. Food Prod. Process. Nutr..

[4] Hadjipanayiotou M. (1995). Composition of ewe, goat and cow milk and of colostrum of ewes and goats. Small Rumin. Res..

[5] Abdelrahman M., Liu G., Al-Saeed F.A., Liu Y., Hou F., Yang H., Farooq U., Ahmed S., Jiang X. (2025). Deciphering the colostral-immunity transfer: From mammary gland to neonates small intestine. Vet. Res. Commun..

[6] Agenbag B., Swinbourne A.M., Petrovski K., van Wettere W.H.E.J. (2021). Lambs need colostrum: A review. Livest. Sci..

[7] Farooq U., Ahmed S., Liu G., Jiang X., Yang H., Ding J., Ali M. (2024). Biochemical properties of sheep colostrum and its potential benefits for lamb survival: A review. Anim. Biotechnol..

[8] Flis Z., Molik E., Ptak A., Szatkowski P. (2025). Natural Bioactive Compounds in Sheep Milk: Potential Biomedical Applications. Curr. Issues Mol. Biol..

[9] Alves A.C., Alves N.G., Ascari I.J., Junqueira F.B., Coutinho A.S., Lima R.R., Perez J.R., De Paula S.O., Furusho-Garcia I.F., Abreu L.R. (2015). Colostrum composition of Santa Ines sheep and passive transfer of immunity to lambs. J. Dairy Sci..

[10] Abuelo A., Cullens F., Hanes A., Brester J.L. (2021). Impact of 2 Versus 1 Colostrum Meals on Failure of Transfer of Passive Immunity, Pre-Weaning Morbidity and Mortality, and Performance of Dairy Calves in a Large Dairy Herd. Animals.

[11] Kelly R.F., Jennings A., Burrough E., Russell G., Adam K., Gascoigne E., Davies P.L., Duncan J.S., Hopker A., Hyde R.M. (2025). Influence of ewe metabolic status on failure of passive transfer of immunity and lamb production in a UK lowland flock. Vet. Rec..

[12] Renaud D.L., Waalderbos K.M., Beavers L., Duffield T.F., Leslie K.E., Windeyer M.C. (2020). Risk factors associated with failed transfer of passive immunity in male and female dairy calves: A 2008 retrospective cross-sectional study. J. Dairy Sci..

[13] McGuire T.C., Regnier J., Kellom T., Gates N.L. (1983). Failure in passive transfer of immunoglobulin G1 to lambs: Measurement of immunoglobulin G1 in ewe colostrums. Am. J. Vet. Res..

[14] Cao X., Ren Y., Lu Q., Wang K., Wu Y., Wang Y., Zhang Y., Cui X.S., Yang Z., Chen Z. (2022). Lactoferrin: A glycoprotein that plays an active role in human health. Front. Nutr..

[15] Navarro R., Paredes J.L., Tucto L., Medina C., Angles-Yanqui E., Nario J.C., Ruiz-Cabrejos J., Quintana J.L., Turpo-Espinoza K., Mejia-Cordero F. (2023). Bovine lactoferrin for the prevention of COVID-19 infection in health care personnel: A double-blinded randomized clinical trial (LF-COVID). Biometals.

[16] Niaz B., Saeed F., Ahmed A., Imran M., Maan A.A., Khan M.K.I., Tufail T., Anjum F.M., Hussain S., Suleria H.A.R. (2019). Lactoferrin (LF): A natural antimicrobial protein. Int. J. Food Prop..

[17] Playford R.J., Weiser M.J., Marchbank T. (2021). Methods to improve efficacy of orally administered bioactive peptides using bovine colostrum as an exemplar. PLoS ONE.

[18] Wang B., Timilsena Y.P., Blanch E., Adhikari B. (2019). Lactoferrin: Structure, function, denaturation and digestion. Crit. Rev. Food Sci. Nutr..

[19] Habib H.M., Ibrahim S., Zaim A., Ibrahim W.H. (2021). The role of iron in the pathogenesis of COVID-19 and possible treatment with lactoferrin and other iron chelators. Biomed. Pharmacother. = Biomed. Pharmacother..

[20] Xu S., Yang Q., Wang R., Tian C., Ji Y., Tan H., Zhao P., Kaplan D.L., Wang F., Xia Q. (2022). Genetically engineered pH-responsive silk sericin nanospheres with efficient therapeutic effect on ulcerative colitis. Acta Biomater..

[21] Li B., Zhang B., Liu X., Zheng Y., Han K., Liu H., Wu C., Li J., Fan S., Peng W. (2022). The effect of lactoferrin in aging: Role and potential. Food Funct..

[22] Moreno-Exposito L., Illescas-Montes R., Melguizo-Rodriguez L., Ruiz C., Ramos-Torrecillas J., de Luna-Bertos E. (2018). Multifunctional capacity and therapeutic potential of lactoferrin. Life Sci..

[23] Kellermayer R., Zilbauer M. (2020). The Gut Microbiome and the Triple Environmental Hit Concept of Inflammatory Bowel Disease Pathogenesis. J. Pediatr. Gastroenterol. Nutr..

[24] Triantaphyllopoulos K.A., Ragia N.D., Panagiotopoulou M.E., Sourlingas T.G. (2025). Integrating Inflammatory and Epigenetic Signatures in IBD-Associated Colorectal Carcinogenesis: Models, Mechanisms, and Clinical Implications. Int. J. Mol. Sci..

[25] Todaro M., Maniaci G., Gannuscio R., Pampinella D., Scatassa M.L. (2023). Chemometric Approaches to Analyse the Composition of a Ewe’s Colostrum. Animals.

[26] Csapó J., Csapó-Kiss Z., Martin T.G., Szentpeteri J., Wolf G. (1994). Composition of colostrum from goats, ewes and cows producing twins. Int. Dairy J..

[27] El-Loly M.M. (2022). Colostrum ingredients, its nutritional and health benefits—An overview. Clin. Nutr. Open Sci..

[28] Moatsou G., Sakkas L. (2019). Sheep milk components: Focus on nutritional advantages and biofunctional potential. Small Rumin. Res..

[29] Durmaz C., Dikme M., Akan E., Bakry A.M. (2025). Colostrum composition of Karya and Çine Çaparı sheep: Variations in fatty acid and protein profiles during early lactation. Int. J. Dairy Technol..

[30] Guiso M.F., Battacone G., Canu L., Deroma M., Langasco I., Sanna G., Tsiplakou E., Pulina G., Nudda A. (2022). Essential and Toxic Mineral Content and Fatty Acid Profile of Colostrum in Dairy Sheep. Animals.

[31] Martini M., Altomonte I., Salari F. (2012). The lipid component of Massese ewes’ colostrum: Morphometric characteristics of milk fat globules and fatty acid profile. Int. Dairy J..

[32] Pavlíková E., Blaško J., Górová R., Addová G., Kubinec R., Margetín M., Soják L. (2010). Variation in fatty acid composition of ewes’ colostrum and mature milk fat. Int. Dairy J..

[33] Navarro F., Galan-Malo P., Pérez M.D., Abecia J.-A., Mata L., Calvo M., Sánchez L. (2018). Lactoferrin and IgG levels in ovine milk throughout lactation: Correlation with milk quality parameters. Small Rumin. Res..

[34] Zhang X., Liu X., Li F., Yue X. (2020). The Differential Composition of Whey Proteomes in Hu Sheep Colostrum and Milk during Different Lactation Periods. Animals.

[35] DiGiacomo K., González-Cabrera M., Capdevila Ospina K., Argüello A., Zamuner F., Castro N. (2026). Factors influencing small ruminant colostrum quality: A comprehensive review from 2020 to present. Small Rumin. Res..

[36] Puppel K., Golebiewski M., Grodkowski G., Slosarz J., Kunowska-Slosarz M., Solarczyk P., Lukasiewicz M., Balcerak M., Przysucha T. (2019). Composition and Factors Affecting Quality of Bovine Colostrum: A Review. Animals.

[37] Vyas S., Kumar M., Patel A.K., Patidar M., Arora K., Kachhawaha S. (2026). Nutritional and biochemical composition of colostrum and milk in sheep of the arid Region. Indian J. Anim. Sci..

[38] Banos G., Clark E.L., Bush S.J., Dutta P., Bramis G., Arsenos G., Hume D.A., Psifidi A. (2019). Genetic and genomic analyses underpin the feasibility of concomitant genetic improvement of milk yield and mastitis resistance in dairy sheep. PLoS ONE.

[39] Ipharraguerre I.R., Rosales Cavaglieri L.A., Rimbach G. (2025). Bioenergetic signatures of circulating blood cells as biosensors of metabolic health in food-producing animals. Front. Vet. Sci..

[40] Conte G., Palombo V., Serra A., Correddu F., D’Andrea M., Macciotta N.P.P., Mele M. (2022). Study of the Fatty Acid Profile of Milk in Different Sheep Breeds: Evaluation by Multivariate Factorial Analysis. Animals.

[41] Sinanoglou V.J., Koutsouli P., Fotakis C., Sotiropoulou G., Cavouras D., Bizelis I. (2015). Assessment of lactation stage and breed effect on sheep milk fatty acid profile and lipid quality indices. Dairy Sci. Technol..

[42] Yakan A., Ozkan H., Camdeviren B., Kaya U., Karaaslan I., Dalkiran S. (2021). Expression patterns of major genes in fatty acid synthesis, inflammation, oxidative stress pathways from colostrum to milk in Damascus goats. Sci. Rep..

[43] Bondoc O., Del Rosario N.A., Del Rosario L.L.G.M., Ramirez D.N. (2023). Fatty Acid Composition and Nutritional Indices/Ratios of Colostrum and Milk from Crossbred Goats in the Philippines. Trop. Anim. Sci. J..

[44] Contarini G., Povolo M., Pelizzola V., Monti L., Bruni A., Passolungo L., Abeni F., Degano L. (2014). Bovine colostrum: Changes in lipid constituents in the first 5 days after parturition. J. Dairy Sci..

[45] Conte G., Foggi G., Tognocchi M., Malucchi G., Tinagli S., Casarosa L., Silvi A., Turini L., Mantino A., Neglia G. (2026). A multivariate approach to exploring interrelationships among milk fatty acids across ruminant species. J. Dairy Sci..

[46] Lou X., Li J., Zhang X., Wang J., Wang C. (2018). Variations in fatty acid composition of Laoshan goat milk from partum to 135 days postpartum. Anim. Sci. J. = Nihon Chikusan Gakkaiho.

[47] Marounek M., Pavlata L., Misurova L., Volek Z., Dvorak R. (2012). Changes in the composition of goat colostrum and milk fatty acids during the first month of lactation. Czech J. Anim. Sci..

[48] Sinanoglou V.J., Cavouras D., Boutsikou T., Briana D.D., Lantzouraki D.Z., Paliatsiou S., Volaki P., Bratakos S., Malamitsi-Puchner A., Zoumpoulakis P. (2017). Factors affecting human colostrum fatty acid profile: A case study. PLoS ONE.

[49] Lunesu M.F., Bomboi G.C., Marzano A., Comin A., Prandi A., Sechi P., Nicolussi P.S., Decandia M., Manca C., Atzori A.S. (2021). Metabolic and hormonal control of energy utilization and partitioning from early to mid lactation in Sarda ewes and Saanen goats. J. Dairy Sci..

[50] Jorjong S., Knegsel A.T.M., Verwaeren J., Lahoz M., Bruckmaier R.M., De Baets B., Kemp B., Fievez V. (2014). Milk fatty acids as possible biomarkers to early diagnose elevated concentrations of blood plasma nonesterified fatty acids in dairy cows. J. Dairy Sci..

[51] Mu T., Hu H., Ma Y., Feng X., Zhang J., Gu Y. (2021). Regulation of Key Genes for Milk Fat Synthesis in Ruminants. Front. Nutr..

[52] Ulbricht T.L., Southgate D.A. (1991). Coronary heart disease: Seven dietary factors. Lancet.

[53] Dietschy J.M. (1998). Dietary fatty acids and the regulation of plasma low density lipoprotein cholesterol concentrations. J. Nutr..

[54] Nantapo C.T., Muchenje V., Hugo A. (2014). Atherogenicity index and health-related fatty acids in different stages of lactation from Friesian, Jersey and FriesianxJersey cross cow milk under a pasture-based dairy system. Food Chem..

[55] Yang C., Du M., Ahmad A.A., Cheng Y., Gebeyew K. (2025). Passive Immunity Establishment Through Colostral IgG Absorption in Neonatal Ruminants: Foundation for Efficient Ruminant Production. Animals.

[56] Ruiz-Larranaga O., Langa J., Rendo F., Manzano C., Iriondo M., Estonba A. (2018). Genomic selection signatures in sheep from the Western Pyrenees. Genet. Sel. Evol. GSE.

[57] Fernández G., Baro J.A., de la Fuente L.F., San Primitivo F. (1997). Genetic Parameters for Linear Udder Traits of Dairy Ewes. J. Dairy Sci..

[58] Gutiérrez-Gil B., El-Zarei M.F., Alvarez L., Bayón Y., de la Fuente L.F., San Primitivo F., Arranz J.J. (2008). Quantitative Trait Loci Underlying Udder Morphology Traits in Dairy Sheep. J. Dairy Sci..

[59] Legarra A., Ugarte E. (2005). Genetic Parameters of Udder Traits, Somatic Cell Score, and Milk Yield in Latxa Sheep. J. Dairy Sci..

[60] Seykora A.J., McDaniel B.T. (1985). Udder and teat morphology related to mastitis resistance: A review. J. Dairy Sci..

[61] Vasileiou N.G., Gougoulis D.A., Riggio V., Ioannidi K.S., Chatzopoulos D.C., Mavrogianni V.S., Petinaki E., Fthenakis G.C. (2018). Association of subclinical mastitis prevalence with sheep breeds in Greece. J. Dairy Res..

[62] Piras C., Ceniti C., Hartmane E., Costanzo N., Morittu V.M., Roncada P., Britti D., Cramer R. (2020). Rapid Liquid AP-MALDI MS Profiling of Lipids and Proteins from Goat and Sheep Milk for Speciation and Colostrum Analysis. Proteomes.

[63] Ait El Alia O., Chaji S., Boukrouh S., Kaddouri M., Zine-Eddine Y., Boutoial K. (2026). Recent advances in the authentication of goat milk and its derived products. J. Food Compos. Anal..

[64] Duliński R., Gancarz M., Shakhovska N., Byczyński Ł. (2025). The Application of Fourier Transform Infrared Spectroscopy and Chemometrics in Identifying Signatures for Sheep’s Milk Authentication. Processes.

[65] Guerrero L., Lamas J.R., Mata L., Razquin P., Corrales F., Paradela A. (2026). Qualitative and quantitative analysis of mixed-milk cheeses by targeted proteomics. J. Dairy Sci..

[66] Hurley W.L., Theil P.K. (2011). Perspectives on immunoglobulins in colostrum and milk. Nutrients.

[67] Mafra I., Honrado M., Amaral J.S. (2022). Animal Species Authentication in Dairy Products. Foods.

[68] Winiarska-Mieczan A., Kwiecień M., Kwiatkowska K., Baranowska-Wójcik E., Szwajgier D., Zaricka E. (2020). Fatty acid profile, antioxidative status and dietary value of the breast muscle of broiler chickens receiving glycine-Zn chelates. Anim. Prod. Sci..

[69] Xu M., Ma B., Zhu K., Tu W., Li C., Hao P., Zhang M. (2026). Research Progress in Multi-Omics Analysis of Dairy Products: Nutritional Quality, Safety Evaluation, and Health Functions. Foods.

[70] Zhao C., Chen N., Ashaolu T.J. (2023). Prebiotic and modulatory evidence of lactoferrin on gut health and function. J. Funct. Foods.

[71] Triantaphyllopoulos K.A. (2023). Long Non-Coding RNAs and Their “Discrete” Contribution to IBD and Johne’s Disease-What Stands out in the Current Picture? A Comprehensive Review. Int. J. Mol. Sci..

[72] Gonzalez-Chavez S.A., Arevalo-Gallegos S., Rascon-Cruz Q. (2009). Lactoferrin: Structure, function and applications. Int. J. Antimicrob. Agents.

[73] Telang S. (2018). Lactoferrin: A Critical Player in Neonatal Host Defense. Nutrients.

[74] Singh A., Malla W.A., Kumar A., Jain A., Thakur M.S., Khare V., Tiwari S.P. (2023). Review: Genetic background of milk fatty acid synthesis in bovines. Trop. Anim. Health Prod..

[75] Tian Z., Zhang Y., Zhang H., Sun Y., Mao Y., Yang Z., Li M. (2022). Transcriptional regulation of milk fat synthesis in dairy cattle. J. Funct. Foods.

[76] Li Z., Zhao X., Jian L., Wang B., Luo H. (2023). Rumen microbial-driven metabolite from grazing lambs potentially regulates body fatty acid metabolism by lipid-related genes in liver. J. Anim. Sci. Biotechnol..

[77] Mierlita D., Daraban S., Lup F. (2011). Effects of breed on milk fatty acid profile in dairy ewes, with particular reference to cis-9, trans-11 conjugated linoleic acid. South Afr. J. Anim. Sci..

[78] Wilms J.N., Hare K.S., Fischer-Tlustos A.J., Vahmani P., Dugan M.E.R., Leal L.N., Steele M.A. (2022). Fatty acid profile characterization in colostrum, transition milk, and mature milk of primi- and multiparous cows during the first week of lactation. J. Dairy Sci..

[79] Settachaimongkon S., Homyog K., Mekboonsonglarp W., Soonoue P., Lerdamnuaylarp T., Prayoonpeeraput P., Theil P.K., Nuntapaitoon M. (2023). Dynamics of fatty acid and non-volatile polar metabolite profiles in colostrum and milk depending on the lactation stage and parity number of sows. Sci. Rep..

[80] Vahmani P., Mapiye C., Prieto N., Rolland D.C., McAllister T.A., Aalhus J.L., Dugan M.E. (2015). The scope for manipulating the polyunsaturated fatty acid content of beef: A review. J. Anim. Sci. Biotechnol..

[81] Chen J., Liu H. (2020). Nutritional Indices for Assessing Fatty Acids: A Mini-Review. Int. J. Mol. Sci..

[82] Monteiro M., Matos E., Ramos R., Campos I., Valente L.M.P. (2018). A blend of land animal fats can replace up to 75% fish oil without affecting growth and nutrient utilization of European seabass. Aquaculture.

[83] Omri B., Chalghoumi R., Izzo L., Ritieni A., Lucarini M., Durazzo A., Abdouli H., Santini A. (2019). Effect of Dietary Incorporation of Linseed Alone or Together with Tomato-Red Pepper Mix on Laying Hens’ Egg Yolk Fatty Acids Profile and Health Lipid Indexes. Nutrients.

[84] Bobe G., Zimmerman S., Hammond E.G., Freeman A.E., Porter P.A., Luhman C.M., Beitz D.C. (2007). Butter composition and texture from cows with different milk fatty acid compositions fed fish oil or roasted soybeans. J. Dairy Sci..

[85] Bonanno A., Di Grigoli A., Mazza F., De Pasquale C., Giosue C., Vitale F., Alabiso M. (2016). Effects of ewes grazing sulla or ryegrass pasture for different daily durations on forage intake, milk production and fatty acid composition of cheese. Animal.

[86] Giorgio D., Di Trana A., Di Napoli M.A., Sepe L., Cecchini S., Rossi R., Claps S. (2019). Comparison of cheeses from goats fed 7 forages based on a new health index. J. Dairy Sci..

[87] Hadrova S., Sedlakova K., Krizova L., Malyugina S. (2021). Alternative and Unconventional Feeds in Dairy Diets and Their Effect on Fatty Acid Profile and Health Properties of Milk Fat. Animals.

[88] Akintola S.L. (2015). Effects of smoking and sun-drying on proximate, fatty and amino acids compositions of Southern pink shrimp (Penaeus notialis). J. Food Sci. Technol..

[89] Potocnik D., Strojnik L., Eftimov T., Levart A., Ogrinc N. (2020). Fatty Acid and Stable Carbon Isotope Composition of Slovenian Milk: Year, Season, and Regional Variability. Molecules.

[90] Yurchenko S., Sats A., Tatar V., Kaart T., Mootse H., Joudu I. (2018). Fatty acid profile of milk from Saanen and Swedish Landrace goats. Food Chem..

[91] Schneider C.A., Rasband W.S., Eliceiri K.W. (2012). NIH Image to ImageJ: 25 years of image analysis. Nat. Methods.

[92] Massouras T., Triantaphyllopoulos K.A., Theodossiou I. (2017). Chemical composition, protein fraction and fatty acid profile of donkey milk during lactation. Int. Dairy J..

[93] Pedregosa F., Varoquaux G., Gramfort A., Michel V., Thirion B., Grisel O., Blondel M., Prettenhofer P., Weiss R., Dubourg V. (2011). Scikit-learn: Machine Learning in Python. J. Mach. Learn. Res..

[94] Tanaka T. (2023). Basics 5. Python+scikit-learn to try with medical image machine learning. Jpn. J. Radiol. Technol..

[95] Tang D., Chen M., Huang X., Zhang G., Zeng L., Wu S., Wang Y. (2023). SRplot: A free online platform for data visualization and graphing. PLoS ONE.

