# Supplementary material for: Biotechnological Advancements in Ovine Bioactives: Mapping Protein Expression Trends and Immuno-Lipidomic Dynamics for Functional Food Applications
Source: Int J Mol Sci. 2026 Aug 14;27(16):7252. doi: 10.3390/ijms27167252 (PMC13513135; doi:10.3390/ijms27167252)
Supplement: Supplementary file 1 [file ijms-27-07252-s001.zip › Supplementary_Material.pdf]

Biotechnological Advancements in Ovine Bioactives: Mapping Protein Expression Trends and Immuno-Lipidomic Dynamics for Functional Food Applications

Kostas A. Triantaphyllopoulos[1]*, Kyriakos Bastas [2], Nikos Moustridis[2], Maria Masoura[2], Theofilos Massouras[2]

[1]Department of Biotechnology, School of Applied Biology and Biotechnology, Agricultural University of Athens, 75 Iera Odos Street, 11855 Athens, Greece
[2]Laboratory of Dairy Research, Department of Food Science and Nutrition, Agricultural University of Athens, 75 Iera Odos Street, 11855 Athens, Greece

* Corresponding author: Department of Biotechnology, School of Applied Biology and Biotechnology, Agricultural University of Athens, 75 Iera Odos St. 11855 Athens, Greece, e-mail: ktrianta@aua.gr, **ORCID**: https://orcid.org/0000-0001-5516-5126

This **supplementary material** file provides additional data matrices and visualizations validating the primary manuscript. These longitudinal colostral expression profiles establish a robust, non-invasive diagnostic framework for translational veterinary medicine, while simultaneously identifying promising natural bioactive targets for human health supplements and immune-modulating functional superfoods. **Specifically, the file contains:**

TABLE OF CONTENTS **page**

Tables
Table S1........ 2
Table S2........ 3
Table S3........ 4
Table S4a........5
Table S4b........6
Table S5........7

Figures
Figure S1........11
Figure S2 12

TABLES

Table S1. Principal Component Analysis (PCA) eigenvalue extraction and variance partition matrix for Chios (n = 5) and Karagouniko (n = 10) longitudinal post-partum IgG ELISA profiles.

Breed Genotype	Principal Component	Eigenvalue (Variance)	Proportion of Variance (%)	Cumulative Variance (%)
**Chios**	**PC1**	3.5571	71.14	71.14
	**PC2**	1.0289	20.58	91.72
	**PC3**	0.4048	8.1	99.82
	**PC4**	0.0092	0.18	100
	**PC5**	0	0	100
**Karagouniko**	**PC1**	3.5855	71.71	71.71
	**PC2**	0.8876	17.75	89.46
	**PC3**	0.4332	8.66	98.13
	**PC4**	0.0874	1.75	99.87
	**PC5**	0.0063	0.13	100

Table S2. Principal component loading coefficients (rotation matrix weights) across all five extracted principal components for individual sampling intervals for Chios (n = 5) and Karagouniko (n = 10) IgG cohorts.

Breed Cohort	IgG Days Post Partum (DPP)	PC1	PC2	PC3	PC4	PC5
**Chios**	**1 (Colostrum)**	0.487	0.125	-0.5887	0.0735	-0.6287
	**3**	0.5195	0.028	-0.3027	-0.4846	0.6347
	**5**	0.4656	-0.2075	0.6724	-0.4006	-0.3571
	**9**	0.522	-0.0725	0.2219	0.7738	0.2726
	**15 (Milk)**	0.0611	0.9671	0.2458	-0.0234	0.0067
**Karagouniko**	**1 (Colostrum)**	0.226	0.9582	0.0058	-0.1507	0.0902
	**3**	-0.4279	0.1258	0.871	0.0776	-0.1909
	**5**	-0.5067	0.02	-0.236	-0.7906	-0.2492
	**9**	-0.5249	0.0614	-0.0921	0.1013	0.8379
	**15 (Milk)**	-0.4834	0.2489	-0.421	0.5796	-0.4374

Table S3. Principal Component Analysis (PCA) eigenvalue extraction and variance partition matrix for Chios (n = 5) and Karagouniko (n = 10) longitudinal post-partum colostrum fatty acid profiles

Breed Genotype	Principal Component	Eigenvalue (Variance)	Proportion of Variance (%)	Cumulative Variance (%)
**Chios**	**PC1**	10.6568	53.28	53.28
	**PC2**	6.0435	30.22	83.5
	**PC3**	2.774	13.87	97.37
	**PC4**	0.5257	2.63	100
	**PC5**	0	0	100
**Karagouniko**	**PC1**	11.4155	57.08	57.08
	**PC2**	4.0482	20.24	77.32
	**PC3**	2.4611	12.31	89.63
	**PC4**	2.0752	10.38	100
	**PC5**	0	0	100

Table S4a. Principal component loading coefficients (rotation matrix weights) for the primary fatty acid variables in Chios breed, determining spatial distribution in the multivariate models.

Fatty Acid	PC1	PC2	PC3	PC4	PC5
C4:0	0.0174	-0.3278	-0.318	-0.3565	-0.1983
C6:0	0.2041	-0.2453	0.2441	0.2285	-0.001
C8:0	-0.0457	-0.3809	0.1895	-0.0537	-0.1666
C10:0	-0.0715	-0.3623	0.2311	-0.0903	0.0241
C12:0	-0.294	-0.1058	-0.0252	0.1333	0.0084
C14:0	-0.2798	0.1639	-0.0302	0.0344	-0.1364
C16:0	-0.2932	0.0981	0.0797	0.1236	-0.3113
C16:1t	-0.183	0.3234	-0.0391	-0.1131	-0.1603
C16:1c	-0.2749	0.0362	-0.1854	-0.4173	-0.1313
C17:0	0.1495	0.3416	0.1091	-0.2128	0.051
C18:0	0.2247	-0.2684	-0.0547	-0.1867	0.0106
C18:1	0.28	-0.1173	-0.1541	0.1707	0.1789
C18:2 n-6	-0.0251	-0.1222	-0.5677	-0.1297	0.2081
C18:3 n-3	0.1386	0.1086	0.4895	-0.3358	-0.0902
CLA	0.2908	0.1116	-0.0267	0.2032	0.0985
SFA	-0.2868	-0.138	0.053	-0.0313	0.1013
MUFA	0.2791	-0.0638	-0.2201	0.1458	-0.4298
PUFA	0.2743	0.0663	0.0896	-0.5332	0.1637
MC SFA	-0.1267	-0.3473	0.189	-0.0408	-0.2004
LC SFA	-0.2899	-0.1129	0.0997	-0.0065	0.645

Table S4b. Principal component loading coefficients (rotation matrix weights) for the primary fatty acid variables in Karagouniko breed, determining spatial distribution in the multivariate models.

Fatty Acid	PC1	PC2	PC3	PC4	PC5
C4:0	0.2066	0.0703	-0.2282	-0.4192	-0.1644
C6:0	0.2703	-0.0448	-0.1587	0.2147	0.093
C8:0	0.2916	-0.0723	-0.0108	-0.0609	-0.0848
C10:0	0.2831	-0.1364	0.0634	-0.0074	-0.0019
C12:0	0.2615	-0.232	8.00E-04	-0.0238	-0.0574
C14:0	-0.1317	-0.2342	0.4834	0.0476	0.3149
C16:0	-0.1813	0.0418	0.4683	0.1938	-0.5343
C16:1trans	-0.2854	0.0841	-0.0679	-0.1202	-4.00E-04
C16:1cis	-0.1608	0.1205	-0.0667	-0.5532	-0.1892
C17:0	-0.2268	-0.2271	-0.1691	-0.2538	-0.0173
C18:0	0.1396	0.4171	-0.1645	-0.0572	-0.1682
C18:1	-0.2895	0.0873	-0.0395	-0.0643	0.2849
C18:2 n-6	-0.0572	0.3886	-0.0907	0.3994	-0.2354
C18:3 n-3	-0.2095	-0.3473	-0.0574	-0.0372	0.0697
CLA	-0.0722	-0.2271	-0.4974	0.2432	0.0768
SFA	0.2919	0.0418	0.0732	-0.0572	0.2258
MUFA	-0.2887	0.0882	-0.0427	-0.0785	0.093
PUFA	-0.1885	-0.1071	-0.3588	0.3337	-0.0562
MC SFA	0.2837	-0.1381	0.0354	-0.0232	0.0044
LC SFA	0.0386	0.4907	0.0561	0.0178	0.5428

Table S5. STROBE Statement—Checklist of items for observational longitudinal cohort studies

Item No	Checklist item	Location in Manuscript
**TITLE and ABSTRACT**		
**1 (a)**	Indicate the study's design with a commonly used term in the title or abstract.	Abstract (Lines 2–4, 14–18)
**1 (b)**	Provide in the abstract an informative and balanced summary of what was done and what was found.	Abstract (Lines 14–36)
**INTRODUCTION**		
**2**	**Background/Rationale:** Explain the scientific background and rationale for the investigation being reported.	Section 1 (Lines 42–129)
**3**	**Objectives:** State specific objectives, including any prespecified hypotheses.	Section 1 (Lines 106–136)
**METHODS**		
**4**	**Study Design:** Present key elements of study design early in the paper.	Section 4.1 (Lines 15–21, 130–136, 807-823)
**5**	**Setting:** Describe the setting, locations, and relevant dates, including periods of recruitment, exposure, follow-up, and data collection.	Section 4.1 (Lines 898–934)
**6**	**Participants:** Give the eligibility/inclusion criteria, and the sources and methods of selection of participants. Describe methods of follow-up.	Section 3.1.2. & 4.1 (Lines 640-648, 825–842)

**7**	**Variables:** Clearly define all outcomes, exposures, predictors, potential confounders, and effect modifiers.	Section 4.1–4.3 (Lines 137–151, 275–299, 516–536, 853-861, 924–936, 1035–1039)
**8**	**Data Sources/Measurement:** For each variable of interest, give sources of data and details of methods of assessment (measurement).	Section 4.2–4.3 (Lines 862–900, 923–964, 975–995, 996–1011)
**9**	**Bias:** Describe any efforts to address potential sources of bias (e.g., standardized feeding, age/parity matching).	Section 3.1.2., 4.1, 4.2.2. (Lines 640-646, 827–840, 898–923)
**10**	**Study Size:** Explain how the study size was arrived at.	Section 3.1.2, 4- 4.1 (Lines 815-818, 825-827, 898–906); Discussion (Lines 643–648)
**11**	**Quantitative Variables:** Explain how quantitative variables were handled in the analyses.	Section 4.4 - 4.5.3. (Lines 513–533, 958–964, 975–990, 996–1003, 1008–1013, 1032–1038, 1063–1071)
**12a**	**Statistical Methods:** Describe all statistical methods, including those used to control for confounding.	Section 4.5 (Lines 965-995)
**12b**	**Statistical Methods:** Describe any statistical methods, used to examine subgroups and interactions.	Section 4.5 (Lines 971-975, 982-985)
**12c**	**Statistical Methods:** How missing data were addressed.	Section 4.5 (Lines 999-1003, 1064–1068)
**12d**	**Statistical Methods:** If applicable, explain how loss to follow-up was addressed.	Section 4.1. & 4.5.2 (Lines 911–913, 975–979)
**12e**	**Statistical Methods:** Describe any sensitivity analyses.	Section 4.5.3. & 4.5.4. (Lines 1008-1033)
**RESULTS**		

**13**	**Participants:** Report numbers of individuals at each stage of study (e.g., numbers potentially eligible).	Sections 2, 3.1.2., 4 & 4.1 (Lines 642–646, 825–833)
**14**	**Descriptive Data:** Give characteristics of study participants (e.g., parity, health status) and information on exposures and potential confounders.	Section 4.1 (Lines 327–329 (Table 1), 534–539 (Table 2)
**15**	**Outcome Data:** Report outcome events or summary measures over time.	Section 2,2.3 & 2.3.2. Tables 1–3, Figures 1–5, Lines 137–168, 274–300, 311–390, 310–329, 534–561
**16**	**Main Results:** Give unadjusted estimates and, if applicable, confounder-adjusted estimates and their precision (e.g., 95% confidence intervals or SEMs).	Section 2 & 3.1.1 & 3.1.4. (Lines 141–151, 154–160, 283–299, 346–362, 379–409, 537–561); Tables 1–3
**17**	**Other Analyses:** Report other analyses done - e.g., analyses of subgroups and interactions, and sensitivity analyses (e.g., PCA, multivariate chemometrics).	Section 2.2–2.3; Figures 4–5; Lines 184–226, 330–416, 513–576, 1072–1100
**DISCUSSION/ CONCLUSIONS**		
**18**	**Key Results:** Summarise key results with reference to study objectives.	Section 2.3.1–2.3.3 & 5. (Lines 577–589, 1043–1067)
**19**	**Limitations:** Discuss limitations of the study, taking into account sources of potential bias or imprecision (e.g., cohort size n=5, band confirmation).	Section 3.1.4, 3.3.1 - 3.3.2 (Lines 690–694, 833–846, 992–995); Section 4.2.2. (Lines 976–996)
**20**	**Interpretation:** Give a cautious overall interpretation of results considering objectives, limitations, multiplicity of analyses, and results from relevant studies.	Section 3.1–3.2 & 3.3.3 (Lines 594–655, 682–694, 695-741, 739–791, 781–805, 847–895)
**21**	**Generalisability:** Discuss the generalisability (external validity) of the study	Section 3.1.3,3., 3.3. & 5 (Lines 656–680, 781-805, 1068-1077)

	results.	
**OTHER INFORMATION**		
**22**	**Funding:** Give the source of funding and the role of the funders for the present study.	Funding Section (Line 1104)

FIGURES

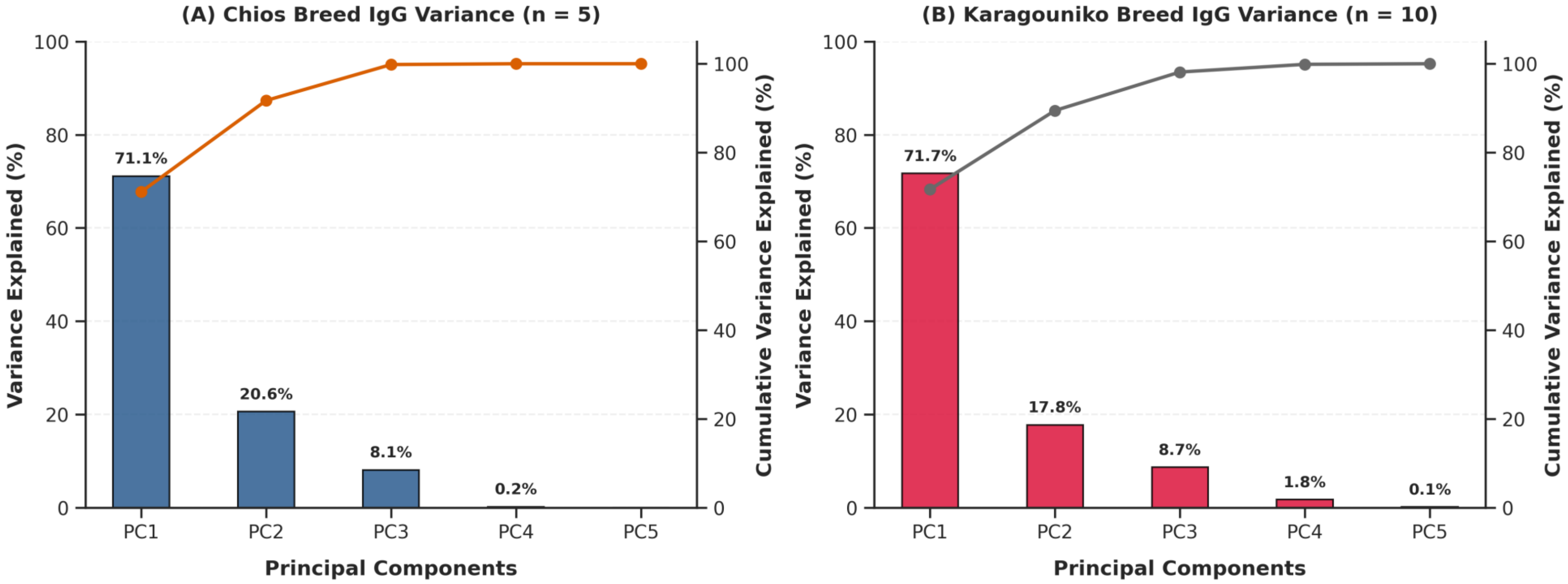

Figure S1. Principal Component Analysis (PCA) explained variance plots illustrating individual and cumulative explained variance percentages for separate longitudinal maternal immunoglobulin G (IgG) ELISA datasets. Panels outline the discrete variance distributions for (A) the dairy Chios cohort (n = 5) and (B) the native indigenous Karagouniko cohort (n = 10). Vertical bar heights show the proportion of variance explained by individual components (PC1–PC5), while secondary line tracks show cumulative totals. The sharp inflection points following PC2 mathematically justify truncating the models to a two-dimensional coordinate system, preserving 91.72% and 89.46% of total operational variance, respectively.

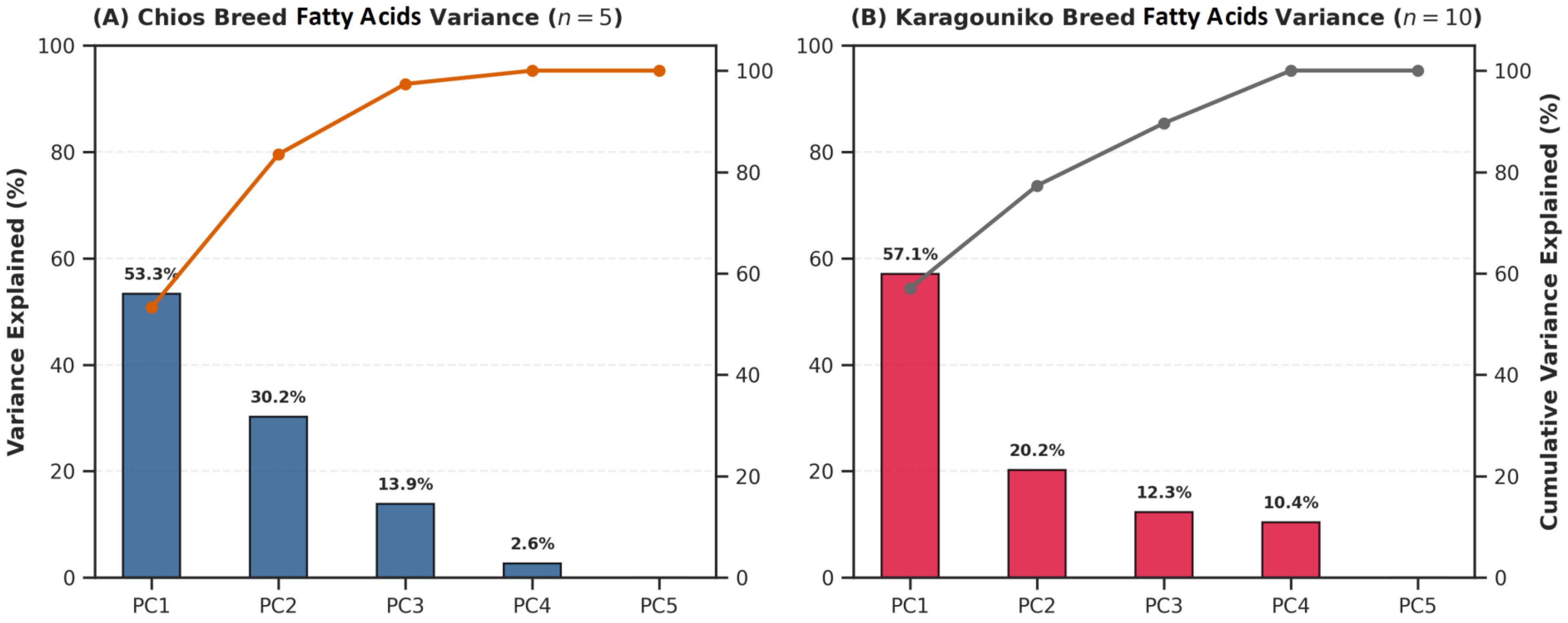

Figure S2. Principal Component Analysis (PCA) explained variance plots illustrating the individual and cumulative explained variance percentages for the first five principal components (PC1–PC5). Panels delineate the distinct mathematical variance distributions derived from longitudinal fatty acid methyl ester (FAME) profiles across the days post-partum transition timeline (DPP 1 to 15) for (A) the high-yielding dairy Chios genotype (n = 5) and (B) the native indigenous Karagouniko genotype (n = 10). The vertical bar heights represent individual component variance percentages (with exact statistical values labeled atop each bar); the secondary upper line tracks represent the cumulative variance threshold. The pronounced structural inflection point ("elbow") visible immediately following PC2 mathematically justifies the truncation of the multivariate models to a two-dimensional subspace, capturing 83.50% and 77.32% of total cumulative experimental variance for the Chios and Karagouniko cohorts, respectively.
